# Integration of digital physicalomics and dual-fluid metabolomics flux ratios reveals tubular secretory dysfunction in early diabetic kidney disease

**DOI:** 10.3389/fendo.2026.1802447

**Published:** 2026-04-22

**Authors:** Haili Zhang, Qian Cai, Jing Gao, Fen Wang, Liping Zhang, Huihui Zhao, Xiaoqiao Ren, Mianzhi Zhang

**Affiliations:** 1 School of Traditional Chinese Medicine, Beijing University of Chinese Medicine, Beijing, China; 2 Institute of Ethnic Medicine, Beijing University of Chinese Medicine, Beijing, China; 3 Department of Nephrology, Dongfang Hospital, Beijing University of Chinese Medicine, Beijing, China; 4 Department of Endocrinology, Third Affiliated Hospital, Beijing University of Chinese Medicine, Beijing, China; 5 Department of Nephrology, Tianjin Hospital of Integrated Traditional Chinese and Western Medicine (ITCWM) Nankai Hospital, Tianjin, China; 6 Nephrology Center, Dongfang Hospital, Beijing University of Chinese Medicine, Beijing, China; 7 Department of Nephrology, Tianjin Institute of Nephropathy of Traditional Chinese Medicine, Tianjin, China

**Keywords:** artificial intelligence, biomarkers, diabetic nephropathies, metabolomics, multicenter study, renal tubular transport

## Abstract

**Background:**

Current screening for diabetic kidney disease (DKD) relies on the estimated glomerular filtration rate (eGFR) and albuminuria, which often fail to detect early tubular dysfunction and non-albuminuric phenotypes. The integration of macroscopic urine physical characteristics with metabolic signatures may offer a novel approach to precision stratification.

**Methods:**

We conducted a multicenter, prospective-retrospective cohort study involving 364 participants with type 2 diabetes. We developed “FluxPro-DKD fusion model,” that integrates “Digital Physicalomics” (computer-vision quantification of urine foam stability and chromaticity) and “Dual-Fluid Metabolomics” (serum-to-urine flux ratios). The model was trained in a discovery cohort (*n*=282) and tested in an independent external validation cohort (*n*=82). The primary outcome was the detection of early-stage DKD. We also assessed the model’s prognostic utility for major adverse renal events over a simulated 3-year period.

**Results:**

Metabolic profiling identified a distinct “serum-to-urine flux mismatch” of protein-bound uremic toxins (e.g., indoxyl sulfate), suggesting tubular secretory failure prior to glomerular damage. Digital physicalomics revealed that urine foam half-life was correlated with albuminuria (*r*=0.78). In the discovery cohort, the FluxPro-DKD fusion model achieved an area under the receiver operating characteristic curve (AUC) of 0.90 (95% confidence interval [CI], 0.87 to 0.93), significantly outperforming the standard clinical model (AUC, 0.78; *P*<0.001). The model maintained robust discrimination in the external validation cohort (AUC, 0.85; 95% CI, 0.79 to 0.91). Among patients with normoalbuminuria, those classified as high-risk by the model had a significantly higher projected 3-year event rate than those classified as low-risk (35.3% vs. 2.3%).

**Conclusions:**

The integration of digital urine physical phenotypes and metabolic flux ratios effectively reveals early tubular secretory dysfunction and improved risk stratification for diabetic kidney disease compared with standard clinical metrics.

## 1 Introduction

Diabetic kidney disease (DKD) remains the leading cause of end-stage renal disease (ESRD) worldwide, affecting approximately 40% of patients with type 2 diabetes mellitus (T2DM) and imposing a staggering burden on global healthcare systems (1, 2). Despite the advent of renoprotective therapies, such as sodium-glucose cotransporter-2 (SGLT2) inhibitors and non-steroidal mineralocorticoid receptor antagonists, the residual risk of progression to kidney failure remains unacceptably high (3). The current clinical paradigm for diagnosing and staging DKD relies almost exclusively on estimated glomerular filtration rate (eGFR) and the urine albumin-to-creatinine ratio (UACR) (4). However, these traditional markers possess inherent limitations: eGFR is a “lagging indicator” that often changes only after significant histological damage has occurred, while albuminuria is subject to high biological variability and fails to capture the increasingly prevalent phenotype of non-albuminuric DKD (5, 6). Consequently, a “diagnostic blind spot” exists in the early stages of the disease, necessitating the discovery of novel, sensitive, and mechanistically insightful biomarkers to enable precision stratification and early intervention.

The kidney functions not merely as a filtration unit but as a highly active metabolic organ, regulating the homeostasis of small molecules through complex glomerular filtration, tubular reabsorption, and secretion processes (7). Therefore, perturbations in renal function are inevitably imprinted on the metabolome of both blood and urine. High-throughput metabolomics has emerged as a powerful tool to decode these molecular signatures (8). While previous studies have identified isolated metabolic markers in either serum or urine, few have integrated these two biological fluids to evaluate the dynamic “crosstalk” between systemic metabolic load and renal handling capacity (9). Recent advances in renal metabolomics have increasingly demonstrated that relative changes between paired serum and urine metabolites—rather than absolute concentrations alone—provide a more sensitive functional readout of tubular injury and altered renal handling before overt changes in eGFR or albuminuria (10, 11)(DOI: 10.7150/ntno.108320; DOI: 10.1089/met.2024.0038). Building upon these foundational pathway-level interpretations, we hypothesize that the dissociation between serum accumulation and urinary excretion of specific metabolites—quantified as the “Serum-to-Urine Flux Ratio”—may serve as a superior proxy for tubular transporter dysfunction, which often precedes glomerular sclerosis in the pathogenesis of DKD.

Furthermore, biological information is not limited to molecular abundance. Historically, macroscopic physical characteristics of urine—such as color, turbidity, and foam stability—have been utilized in traditional medical systems (e.g., Traditional Chinese Medicine and Tibetan Medicine) to assess kidney health. While often dismissed as subjective or empirical, these macroscopic features are physical manifestations of urine composition (e.g., surface tension changes driven by surfactant proteins). In the era of digital health, the application of computer vision and deep learning offers an unprecedented opportunity to digitize these “soft” phenotypes into objective, quantifiable “Physicalomics” data (12–14). However, the integration of such macroscopic digital phenotypes with microscopic molecular profiles remains an unexplored frontier in nephrology. To bridge these gaps, we conducted a multi-center study integrating Dual-Fluid Metabolomics with computer-vision-based urine phenotyping. We established a prospective-retrospective Discovery Cohort (*n*=282) comprising healthy controls, T2DM patients without kidney disease, and patients with confirmed DKD, alongside an independent External Validation Cohort (*n*=82) to ensure model generalizability. By employing nuclear magnetic resonance (NMR) spectroscopy, we aimed to map the trajectory of metabolic aberrations across the disease spectrum. Concurrently, we developed a novel “Digital Urine” pipeline to quantify chromatic and tensiometric features of urine samples. We hope that this multi-omics fusion approach could enhance early diagnostic accuracy and risk stratification compared to standard clinical metrics, and provide a new dimension for the precision management of diabetic kidney disease.

## 2 Participants and methods

### 2.1 Study design

This study utilized a two-center, prospective-retrospective cohort design comprising a Discovery Cohort for biomarker identification and model training, and an independent External Validation Cohort for evaluating model generalizability. The retrospective phase consisted of extracting historical electronic medical record (EMR) data—specifically prior eGFR and UACR trajectories—to accurately define the chronicity of baseline clinical phenotypes. Conversely, the prospective phase involved the real-time, standardized collection of paired serum and urine samples, alongside immediate digital physicalomics imaging, to acquire the cross-sectional multi-modal data required for model construction.

The study protocol was approved by the Institutional Review Boards (IRB) of the Beijing University of Chinese Medicine Second Affiliated Hospital (Dongfang Hospital) (Ethical approval number: JDF-IRB-2020000601) and Beijing University of Chinese Medicine Third Affiliated Hospital (Ethical approval number: 2024BZYLL1007). The study was conducted in strict accordance with the Declaration of Helsinki. Written informed consent was obtained from all participants prior to inclusion.

### 2.2 Inclusion and exclusion criteria

Participants were recruited from the inpatient and outpatient departments of Dongfang Hospital and the Third Affiliated Hospital of Beijing University of Chinese Medicine between September 2024 and September 2025. Participants were stratified into three groups based on the 2020 Chinese Guidelines for Type 2 Diabetes and the 2023 Expert Consensus on Diabetic Kidney Disease: (1)Healthy Controls (HC, n=80): Individuals recruited from the physical examination center with normal glucose tolerance, normal liver/kidney function, and no history of chronic disease. (2)T2DM without Kidney Disease (T2DM-NoDKD, *n*=102): Patients with a confirmed diagnosis of T2DM, aged 18–80 years, with an estimated Glomerular Filtration Rate (eGFR) ≥ 90 mL/min/1.73m² and normoalbuminuria (UACR< 30 mg/g) on at least two consecutive visits. (3)Diabetic Kidney Disease (DKD, *n*=100): T2DM patients with confirmed DKD, defined as persistent albuminuria (UACR ≥ 30 mg/g) and/or sustained reduction in eGFR (< 60 mL/min/1.73m²) for >3 months, excluding other primary renal pathologies. External Validation Cohort: An independent cohort of 82 participants was recruited from a geographically distinct center to assess the robustness of the predictive models. This cohort followed identical inclusion and exclusion criteria to the Discovery Cohort. Participants were excluded if they met any of the following: (1) Non-diabetic renal diseases (e.g., IgA nephropathy); (2) Acute metabolic complications (e.g., DKA) or acute infection within the last 3 months; (3) Severe organic diseases of the heart, liver, or brain; (4) Pregnancy or lactation; (5) History of psychiatric disorders or drug/alcohol dependence; (6) Intake of medications or foods known to significantly alter urine color (e.g., rifampin, beetroot) within 24 hours prior to sampling.

**Figure 1:**
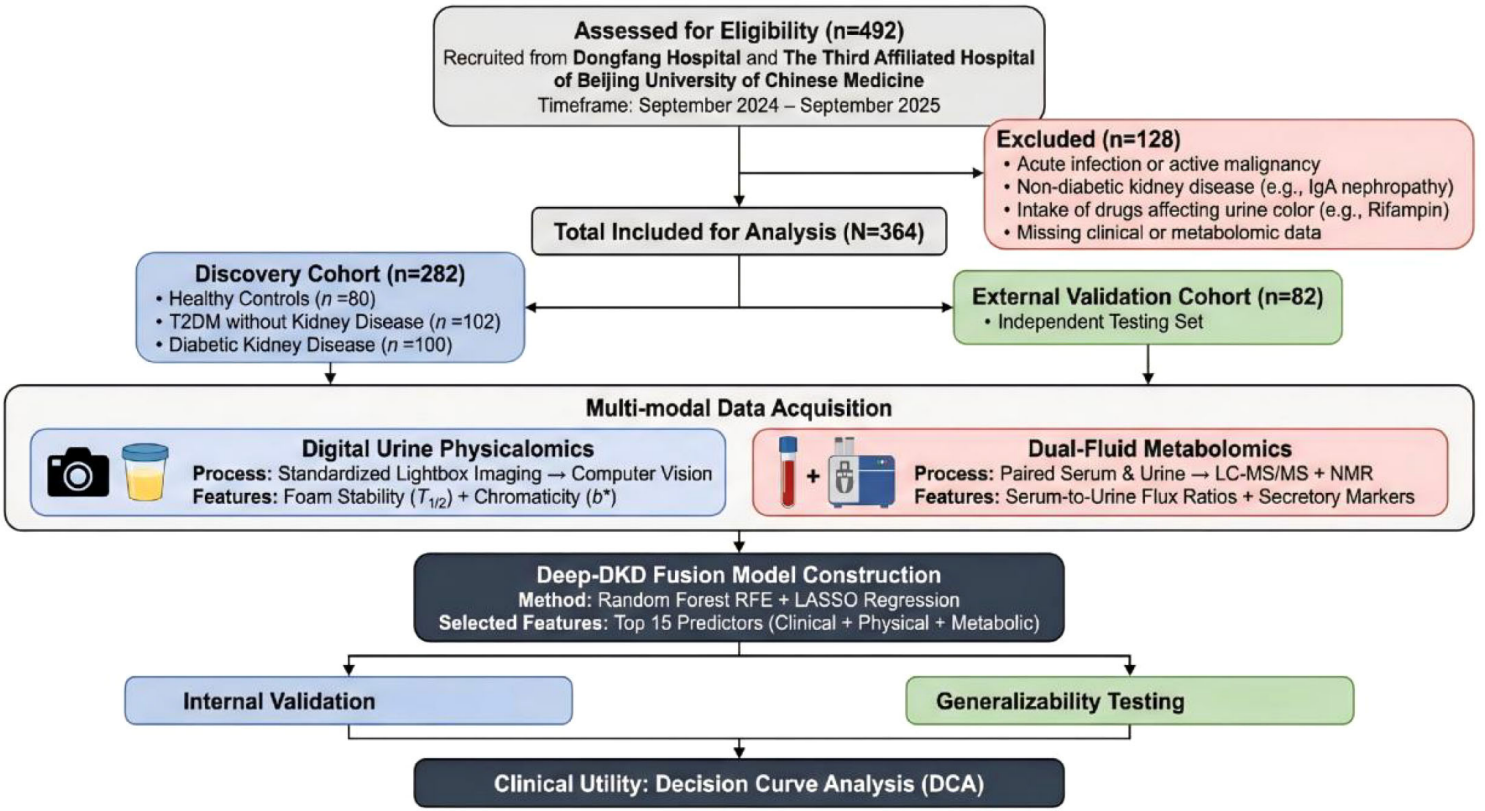
Study participant recruitment and multi-modal analysis workflow. The flowchart illustrates the participant selection process and the study design. Out of 492 screened patients, 364 were included in the final analysis and divided into a Discovery Cohort (*n*=282) and a geographically distinct External Validation Cohort (*n*=82). All participants underwent paired serum and urine sampling for dual-fluid metabolomics and standardized digital urine imaging for physicalomics profiling. The analytical pipeline details the feature selection strategy and the construction of the FluxPro-DKD fusion model. Abbreviations: DKD, Diabetic Kidney Disease; T2DM, Type 2 Diabetes Mellitus; RF-RFE, Random Forest Recursive Feature Elimination; DCA, Decision Curve Analysis.

A total of 492 individuals were initially screened from Dongfang Hospital and The Third Affiliated Hospital of Beijing University of Chinese Medicine between September 2024 and September 2025. After excluding 128 subjects based on strict criteria (e.g., non-diabetic renal disease, confounding medication use), 364 eligible participants were enrolled and stratified into a Discovery Cohort (*n*=282) for model training and an independent External Validation Cohort (*n*=82) for testing (**Figure 1**).

### 2.3 Digital quantification of macroscopic urine phenotypes (urine physicalomics)

We developed a novel, computer vision-assisted pipeline to quantify traditional macroscopic urine features into objective physical phenotypes.

#### 2.3.1 Standardized image acquisition

Urine samples were collected in standard medical cups and immediately transferred to optical-grade square glass cuvettes (10 mm path length) to eliminate curvature distortion. Imaging was performed within a custom-designed light-controlled enclosure equipped with high-CRI (>95) LED arrays providing uniform diffuse lighting at a fixed color temperature of 5500K. An X-Rite ColorChecker Passport was positioned within the field of view for every capture to enable post-hoc white balance and color correction (15).

#### 2.3.2 Computer vision algorithms

##### 2.3.2.1 Chromatic quantification

Raw images were pre-processed for color correction using the reference color card. The Region of Interest (ROI) corresponding to the urine liquid column was segmented. RGB values were converted to the CIELAB color space (*L×a×b*) to mimic human visual perception. Specifically, the b× value (representing the blue-yellow axis) and Chroma (C×) were extracted as quantitative metrics for “urine yellowness,” serving as a digital proxy for urine concentration and potential oxidative stress markers.

##### 2.3.2.2 Foam stability analysis (surfactant-related phenotypes)

To quantify “foamy urine” (a proxy for proteinuria), we implemented a Standardized Agitation Protocol. Samples were subjected to mechanical oscillation (5 Hz for 5 seconds). Time-lapse imaging was triggered immediately upon cessation (*t*=0, 30*s*, 60*s*, 120*s*). OpenCV algorithms were employed to detect foam boundaries and bubble morphology. Two novel features were engineered: 1)Foam Half-life (*T*_1/2_): The time required for the foam layer height to decay by 50%. 2)Micro-bubble Index (MBI): The ratio of the area occupied by micro-bubbles (diameter< 0.5 mm) to the total foam area at *t*=30s.

##### 2.3.2.3 Multi-platform metabolomics profiling

To achieve comprehensive and quantitatively rigorous coverage of the paired serum and urine metabolome, we employed a two-tiered, multi-platform analytical strategy. Initial global untargeted profiling was conducted via high-resolution LC-MS/MS to identify broad pathway perturbations, yielding 845 serum and 612 urine features. Subsequently, for the calculation of Serum-to-Urine Flux Ratios, absolute quantification of a pre-defined 45-metabolite targeted panel was performed using orthogonal platforms tailored to analyte abundance and physicochemical properties. High-abundance, hydrophilic central carbon metabolites (e.g., TCA cycle intermediates, amino acids) were quantified using 1*^H^*-NMR spectroscopy on a Bruker Avance III 600 MHz system equipped with a NOESY pulse sequence and TSP as an internal standard. Conversely, low-abundance, highly protein-bound analytes—specifically uremic retention solutes (Indoxyl Sulfate, p-Cresyl Sulfate) and long-chain acylcarnitines—were exclusively quantified via targeted Triple Quadrupole Mass Spectrometry (QqQ-MS) in Multiple Reaction Monitoring (MRM) mode, utilizing stable isotope-labeled internal standards (SIL-IS) to achieve precise absolute concentrations (MSI Level 1).

##### 2.3.2.4 Targeted quantification of protein-bound uremic toxins

Given their low abundance and high protein-binding affinity, absolute quantification of Indoxyl Sulfate (IS) and p-Cresyl Sulfate (pCS) was achieved exclusively via targeted HPLC-QqQ-MS in MRM mode, rather than 1*^H^*-NMR. To correct for matrix effects and ensure robust quantification across disparate biofluids, samples were spiked with Stable Isotope-Labeled Internal Standards (SIL-IS; ^13^*C*_6_-*IS* and *d*_7_-*pCS*) prior to extraction. Metabolite identification rigorously met the Metabolomics Standards Initiative (MSI) Level 1 criteria, requiring exact matches of chromatographic retention time (*t_R_* ± 0.1 min) and dual MS/MS ion transitions against authentic high-purity reference standards. The analytical Limits of Detection (LOD) and Limits of Quantification (LOQ) were experimentally established (IS: LOD 15.2 nmol/L, LOQ 45.5 nmol/L; pCS: LOD 22.4 nmol/L, LOQ 67.0 nmol/L). All clinical measurements utilized for Serum-to-Urine Flux Ratio calculations fell robustly above the *LOQ* and within the validated linear dynamic range. Detailed mass spectrometry parameters are provided in **Supplementary Table 1**.

##### 2.3.2.5 Standardized agitation and tensiometric validation

The mechanical oscillation parameters (5 Hz for 5 seconds) were established following a systematic preliminary gradient analysis. These parameters were selected to optimize the signal-to-noise ratio for AI segmentation by generating sufficient initial foam volume without inducing unphysiological protein denaturation and shear-induced cross-linking at the air-liquid interface. To validate this optical approach against established biophysical standards, a subset of 40 samples was concurrently analyzed using classical Pendant Drop Tensiometry to measure equilibrium surface tension (*γ*). A robust inverse correlation (Spearman’s *ρ* = -0.88, *P*< 0.001) was observed between true surface tension and the optically derived Foam Half-life (*T_1/2_*), confirming that this standardized mechanical protocol accurately captures the underlying surfactant physics of pathological proteinuria.”

#### 2.3.3 Analytical validation of the digital physicalomics pipeline

To evaluate the clinical viability of the digital physicalomics measurements, analytical precision and agreement were rigorously quantified. Intra-assay repeatability (10 consecutive replicates) and inter-assay intermediate precision (assessed across 5 days by two independent operators) demonstrated excellent stability. The Coefficients of Variation (CV) remained strictly below 4.5% for both Foam Half-life (T1/2) and Chromaticity (b) across all tests. Furthermore, Bland-Altman analysis of paired technical replicates (n=50) confirmed high systemic agreement, yielding a near-zero mean bias (+0.15 s for T1/2; -0.08 for b*) with >95% of measurements falling securely within the clinically acceptable 95% limits of agreement (± 1.96 SD) without proportional bias. Detailed validation protocols and plots are provided in **Supplementary Figure 1**.

### 2.4 Biospecimen collection and preparation

#### 2.4.1 Mathematical derivation of the serum-to-urine flux ratio

To quantitatively assess tubular secretory dysfunction independent of baseline glomerular filtration and hydration status, we computed a double-creatinine-normalized Serum-to-Urine Flux Ratio for all targeted metabolites. To ensure dimensional parity, the absolute concentrations of both the target metabolites and creatinine in matched serum and urine samples were uniformly converted to *μ*mol/L. The dimensionless ratio was calculated using the following equation:


$$Flux\_Ratio_{metabolite}=\log_{10}\left(\frac{{\left[C\right]}_{serum\_metabolite}/{\left[C\right]}_{serum\_creatinine}}{{\left[C\right]}_{urine\_metabolite}/{\left[C\right]}_{urine\_creatinine}}\right)$$

To maintain computational continuity and prevent zero-division errors in the logarithmic transformation, any metabolite concentration strictly falling below the platform-specific Limit of Detection (LOD) was imputed as $\text{LOD/}\sqrt{2}.$ This log_10_-transformed, dimensionless flux signature was subsequently utilized for all downstream multivariable regression models and Random Forest feature selection.

#### 2.4.2 Global normalization and sensitivity analysis

For the untargeted global profiling datasets, initial correction for varying urine dilution was performed using Probabilistic Quotient Normalization (PQN) referenced to the median spectrum of the pooled Quality Control (QC) samples. Because severe metabolic pathologies like DKD may induce asymmetric systemic shifts that violate PQN assumptions, we conducted a rigorous sensitivity analysis. The raw untargeted urine matrix was re-normalized using an orthogonal approach: Creatinine-Adjusted Total Useful Signal (TUS) normalization. Multivariate clustering (PCA/OPLS-DA) and pathway enrichment outputs generated from this alternative normalization matrix were highly congruent (>90% feature overlap) with the PQN-derived results, confirming that the observed global metabolic perturbations are robust biological signatures and not normalization artifacts. (Note: Targeted absolute quantification data utilized for Serum-to-Urine Flux Ratios were strictly independent of global normalization, utilizing internal SIL-IS and direct physiological creatinine correction).

#### 2.4.3 Sample collection

Peripheral venous blood (5 mL) and mid-stream morning urine (40 mL) were collected from all participants after an overnight fast of 8-12 hours. To minimize pre-analytical variability, participants were instructed to avoid tea, alcohol, and dairy products the night before collection. 1)Serum: Blood was allowed to clot, then centrifuged at 3000 rpm (1500 × *g*) for 5 minutes at 4°C (16–18). 2)Urine: Urine was centrifuged at 3000 rpm (1500 × *g*) for 5 minutes at 4°C to remove cellular debris and sediments. 3)Storage: Supernatants were aliquoted (1000 *μ*L/tube) and stored at -80°C until analysis.

To eliminate potential confounders affecting urine surface tension and chromaticity, a rigorous Standard Operating Procedure (SOP) was implemented. All participants adhered to a strict 12-hour overnight fast to minimize postprandial lipuria, which can destabilize foam formation. Participants were specifically instructed to avoid high-fat meals and surfactant-rich foods (e.g., dairy, emulsifiers) for 24 hours prior to sampling. To prevent contamination from exogenous surfactants (e.g., residual detergents), all optical cuvettes were acid-washed (10% HCl), rinsed with deionized water, and dried in a dust-free environment before use. Surface tension is temperature-dependent. All samples were equilibrated to room temperature (22°C ± 1°C) and imaged immediately (<5 minutes) after voiding to prevent bacterial growth or protein degradation. In addition, urine samples with visible turbidity unrelated to crystallization, or signs of contamination (e.g., menstrual blood), were excluded.”

#### 2.4.4 Metabolomics profiling

To achieve comprehensive coverage of the metabolome, we used the Targeted MS/NMR (for quantification). Urine samples (540 *μ*L) were mixed with 60 *μ*L of phosphate buffer (pH 7.4) containing 10% D_2_O and 0.05% TSP (3-trimethylsilyl-propionic-2,2,3,3-d4 acid sodium salt) as a chemical shift reference. Spectra were acquired on a Bruker Avance III 600 MHz spectrometer at 298 K using the standard 1D NOESY pulse sequence (noesypr1d) to suppress the water signal.

Our metabolomics workflow utilized a two-tiered approach. Initially, global untargeted profiling via LC-MS/MS yielded 845 serum and 612 urine features to map broad pathway perturbations. From this broader pool, a fully overlapping panel of 45 pre-defined metabolites—specifically targeting uremic toxins (e.g., IS, pCS) and acylcarnitines—underwent absolute targeted quantification using Triple Quadrupole MS and 1H-NMR with internal standards (TSP). Serum-to-urine flux ratios were calculated exclusively using this targeted, fully overlapping panel to ensure high analytical precision. To address missingness, which can artificially skew Serum-to-Urine Flux Ratios, targeted features with >20% missing values across either biofluid were excluded. For the remaining data, missing values were imputed using a K-Nearest Neighbors (KNN) algorithm prior to the calculation of Serum-to-Urine Flux Ratios.

To eliminate the confounding effect of polyuria and varying urinary concentrations, formal dilution corrections were applied. For chromatic features, the yellowness index (*b*) was mathematically normalized to a physiological baseline using the formula: b*_adj_ = b* × (650/Osmolality_sample_). For non-linear tensiometric features (*T_1/2_*), urine osmolality was incorporated as a mandatory continuous covariate in all partial correlation analyses and multivariable prediction models.

### 2.5 Data processing and statistical analysis

NMR Data were processed using MestReNova 6.0 (Mestrelab Research) for phase and baseline correction. Metabolites were identified using Chenomx Profiler 9.2. To account for urine dilution variability, urine metabolomics data were normalized using Probabilistic Quotient Normalization (PQN) referenced to the median spectrum of the QC samples.

#### 2.5.1 Definition of simulated clinical endpoints

To evaluate the potential prognostic utility of the FluxPro-DKD Fusion Model, we defined a primary composite endpoint of Major Adverse Renal Events (MARE) for use in our simulated projection. MARE comprised a sustained decline in eGFR of ≥ 30% from baseline, the onset of macroalbuminuria (UACR >300 mg/g), or progression to End-Stage Renal Disease (ESRD). Clinical data were censored at the time of the last follow-up visit or the occurrence of the primary event.

#### 2.5.2 Target trial emulation and prognostic projection

To estimate the long-term clinical utility of the FluxPro-DKD fusion model beyond cross-sectional diagnosis, we conducted a Simulated Target Trial analysis. Since long-term follow-up data for the validation cohort is currently accruing, we performed a prognostic projection to simulate 3-year renal outcomes. This emulation applied the hazard ratios (HR) derived from established risk trajectories of similar phenotypes in the Discovery Cohort and external longitudinal datasets.

We defined the primary composite endpoint (MARE) as a sustained 30% decline in eGFR, the onset of macroalbuminuria (UACR >300 mg/g), or progression to End-Stage Renal Disease (ESRD). We assigned prognostic probabilities to each participant in the External Validation Cohort (*n*=82) based on their baseline FluxPro-DKD fusion model risk scores. A Monte Carlo simulation (1,000 iterations) was employed to project the incidence of adverse events and eGFR slope trajectories over a 3-year horizon. This framework allows for the assessment of the model’s potential to stratify “silent progressors” (high-risk T2DM-NoDKD patients) who would otherwise be missed by conventional albuminuria screening.

### 2.6 Sample size and cohort allocation

#### 2.6.1 For the discovery cohort (n=282)

Rather than relying solely on the traditional “rule of thumb” of Events Per Variable (EPV) ≥ 10, we mathematically justified the training sample size based on the criteria required to minimize the global shrinkage factor (*S*) and limit optimism in model fit. For a binary clinical prediction model, the minimum sample size (*n*) required to achieve a target shrinkage factor (typically *S* ≥ 0.9 to prevent overfitting) is calculated using the following equation:


$$n=\frac{p}{\left(S-1)\ln(1-\frac{R_{CS}^{2}}{S}\right)}$$

where *p* represents the number of candidate predictor parameters, and *R^2CS^* is the anticipated Cox-Snell R-squared. Given an anticipated *R^2CS^* of 0.25 (typical for robust biomarker panels) and *p*=15 candidate features evaluated during the Random Forest RFE process, the required minimum sample size to achieve *S* ≥ 0.9 is approximately 255. Our discovery cohort of *n*=282 (with 100 DKD events) comfortably exceeds this mathematical requirement. Furthermore, computing the classical EPV for our final 8-variable nomogram yields an EPV of 12.5 (100/8), which robustly satisfies the standard ≥ 10 threshold.

#### 2.6.2 For the external validation cohort (*n*=82)

For the external validation set, the primary objective is to estimate the Area Under the Receiver Operating Characteristic Curve (AUC) with sufficient precision. The sample size was mathematically justified using the Hanley and McNeil formula for the standard error of the AUC:


$$SE(AUC)=\sqrt{\frac{AUC(1-AUC)+(n_{1}-1)(Q_{1}-AUC^{2})+(n_{2}-1)(Q_{2}-AUC^{2})}{n_{1}n_{2}}}$$

where *n_1_* and *n_2_* are the number of cases and controls, and *Q_1_* and *Q_2_* are distribution-specific probabilities. Assuming a null AUC of 0.5 and an anticipated true AUC of > 0.80, an independent validation cohort of *n*=82 provides greater than 85% statistical power (1-*β* > 0.85, two-sided *α* = 0.05) to detect a significant discriminative ability.

#### 2.6.3 Rationale for the 3:1 cohort allocation

The specific allocation (*n*=282 vs. *n*=82, approximately 3:1) was prospectively determined. This ratio aligns with standard machine learning conventions to maximize the high-dimensional multi-omics data fed into the complex RF-RFE training phase, while strictly reserving a validation set that met the mathematical power thresholds described above for independent AUC verification.

To strictly prevent data leakage and optimistic bias during model construction, feature selection was performed using Random Forest Recursive Feature Elimination (RF-RFE) strictly embedded within a nested 10-fold cross-validation framework. Feature selection was isolated to the inner training loops, ensuring that the hold-out validation folds in the outer loop remained completely blind to the selection process.

### 2.7 Statistical analysis

All statistical analyses were performed using R software (version 4.3.0) and SIMCA 14.1.

Differences between groups were assessed using the Mann-Whitney U test or Kruskal-Wallis test. *P*-values were adjusted for false discovery rate (FDR) using the Benjamini-Hochberg method. Principal Component Analysis (PCA) and Orthogonal Partial Least Squares Discriminant Analysis (OPLS-DA) were employed to identify differential metabolites (VIP > 1.0). Pathway enrichment analysis was performed using MetaboAnalyst 5.0.

We employed a Recursive Feature Elimination (RFE) strategy combined with Random Forest (RF) algorithms to select the most robust predictors. Three models were constructed: (1) Clinical Base Model (Age, BMI, HbA1c, SBP); (2) Metabolomics Enhanced Model (Base + Top Serum/Urine Markers); (3) FluxPro-DKD Fusion Model (Metabolomics + Digital Urine Phenotypes). Performance was evaluated using AUC-ROC, Calibration Plots, and Decision Curve Analysis (DCA). The incremental value was quantified using Net Reclassification Improvement (NRI) and Integrated Discrimination Improvement (IDI) indices.

To validate the Orthogonal Partial Least Squares Discriminant Analysis (OPLS-DA) models and exclude the possibility of overfitting, 1,000-iteration permutation tests were performed for both the serum and urine datasets. A model was considered robust and not overfitted if all permuted *R*^2^ and *Q*^2^ values were lower than the original metrics, and the y-intercept of the *Q*^2^ regression line was less than zero. Internal validation and performance estimation of the machine learning models were conducted using a repeated Stratified 10-fold Cross-Validation approach (5 repeats). Stratification was employed to strictly maintain the clinical prevalence ratio of DKD across all folds. To accurately quantify model optimism, the apparent performance (evaluated on the entire training set) is explicitly reported separately from the cross-validated performance (evaluated strictly on the hold-out folds). To rigorously account for the high incidence of non-renal mortality (primarily cardiovascular events) characteristic of DKD populations, survival analyses were performed using both standard Cox proportional hazards regression and Fine-Gray subdistribution hazard models. Non-renal mortality was formally treated as a competing risk in the Fine-Gray models to ensure that the estimated prognostic utility of our multi-modal signatures was not biased by informative censoring.

### 2.8 Data availability

The untargeted metabolomics raw data have been deposited in the EMBL-EBI MetaboLights database (Accession Number: MTBLS14007). The code used for the ‘Digital Urine’ computer vision analysis is available on GitHub (URL: github.com/HailiZhang0112/FluxPro-DKD).

## 3 Results

### 3.1 Clinical characteristics and digitization of macroscopic urine phenotypes

**Table 1** summarizes the baseline clinical, demographic, and multi-omics characteristics of the 364 study participants, stratified into the discovery cohort (Healthy Controls, *n*=80; T2DM-NoDKD, *n*=102; DKD, *n*=100) and the independent external validation cohort (*n*=82). As expected, compared to healthy controls and T2DM-NoDKD patients, those with established DKD exhibited significantly more advanced disease trajectories, including longer diabetes duration, poorer glycemic control (HbA1c 8.1 ± 1.5%), and profound renal impairment characterized by a depressed eGFR (54.1 ± 18.2 mL/min/1.73m^2^) and pronounced albuminuria (median UACR 482.5 mg/g) (all *P*< 0.001). Crucially, the novel digital urine physicalomics pipeline successfully captured macroscopic phenotypic shifts that paralleled this clinical progression; specifically, DKD patients demonstrated a marked loss of urinary concentrating ability—evidenced by significantly reduced osmolality (420.3 ± 110.6 mOsm/kg) and yellowness index (b* = 24.6 ± 6.5) —alongside a robust tensiometric “surfactant signature” defined by a drastically prolonged foam half-life (*T_1/2_* = 45.8 seconds) and an elevated MBI (18.2 ± 6.5%) compared to non-DKD counterparts (*P*< 0.001). Additionally, comparative analysis confirmed that the external validation cohort was highly analogous to the discovery population, exhibiting comparable baseline age, renal function (eGFR), and albuminuria (UACR) trajectories (*P* > 0.05 for all comparisons). Crucially, to ensure these macroscopic signatures were not merely artifacts of urinary dilution—given the significantly lower osmolality in DKD patients (420.3 mOsm/kg) —we applied strict dilution corrections. Following mathematical normalization for osmolality, the adjusted yellowness index (b*_adj_) remained significantly attenuated in DKD (*P<* 0.001). Furthermore, partial correlation analysis adjusting for urine osmolality confirmed that Foam Half-life (*T_1/2_*) maintained a highly significant, independent association with pathological albuminuria (partial *r* = 0.74, *P<* 0.001), confirming that the digital surfactant signature accurately reflects protein leakage rather than simple variations in water excretion.

**Table 1:** Baseline clinical characteristics of the study populations.

Variable	Healthy controls (*n* = 80)	T2DM-NoDKD (*n* = 102)	DKD (*n* = 100)	External validation (n = 82)	*P*-value
Demographics					
Age (years)	54.2 ± 8.5	58.1 ± 9.2	61.4 ± 8.8	58.6 ± 9.0	< 0.001
Sex (Male), n (%)	42 (52.5%)	55 (53.9%)	58 (58.0%)	45 (54.9%)	0.742
Body Mass Index (kg/m²)	23.1 ± 2.8	26.4 ± 3.5	27.1 ± 3.9	25.8 ± 3.6	< 0.001
SBP (mmHg)	118.2 ± 12.4	129.7 ± 15.0	142.3 ± 18.6	131.2 ± 15.8	< 0.001
DBP (mmHg)	76.3 ± 8.6	80.2 ± 9.3	84.4 ± 10.2	80.5 ± 9.5	< 0.001
Diabetes History					
Duration of Diabetes (years)	—	7.5 (4.0–11.5)	12.0 (8.0–16.5)	9.5 (5.0–13.5)	< 0.001
HbA1c (%)	5.4 ± 0.4	7.2 ± 1.1	8.1 ± 1.5	7.0 ± 1.3	< 0.001
HbA1c (mmol/mol)	36.7 ± 4.2	55.1± 12.7	65.4 ± 16.2	53.4 ± 10.5	< 0.001
Renal Function					
Serum Creatinine (μmol/L)	68.2 (59.3–77.2)	74.0(62.4–85.3)	112.1 (88.6–145.7)	82.3 (64.4–110.9)	< 0.001
eGFR (mL/min/1.73m²)	112.5 ± 11.3	96.2 ± 14.7	54.1 ± 18.2	88.6 ± 22.4	< 0.001
UACR (mg/g)	6.2 (4.1–8.5)	14.5 (8.2–22.1)	482.5 (156.4–1250.2)	42.5 (9.8–215.4)	< 0.001
Hematology Profile					
White Blood Cell Count (109/L)	5.8 ± 1.2	6.4 ± 1.5	6.9 ± 1.8	6.3 ± 1.3	0.032
Hemoglobin (g/L)	142.4 ± 11.3	136.2± 14.6	118.5 ± 16.2	133.8 ± 13.1	< 0.001
Platelet Count (109/L)	245.6 ± 52.3	230.9± 60.7	215.4 ± 65.3	228.6 ± 48.2	0.045
Neutrophil-Lymphocyte Ratio (NLR)	1.8 ± 0.6	2.2 ± 0.8	2.9 ± 1.1	2.3 ± 0.9	< 0.001
Urinalysis Parameters					
Urine Specific Gravity (SG)	1.018 ± 0.004	1.020 ± 0.005	1.012 ± 0.006	1.017 ± 0.005	< 0.001
Urine pH	6.2 ± 0.5	5.6 ± 0.6	5.8 ± 0.7	5.8 ± 0.4	0.015
Urine Osmolality (mOsm/kg)	650.2 ± 120.6	680.1 ± 145.4	420.3± 110.6	585.5 ± 93.3	< 0.001
Lipid Profile					
Total Cholesterol (mmol/L)	4.5 ± 0.8	4.9 ± 1.0	5.6 ± 1.2	5.0 ± 1.1	< 0.001
Triglycerides (mmol/L)	1.2 (0.9–1.6)	1.8 (1.3–2.4)	2.5 (1.8–3.6)	1.8 (1.1–2.5)	< 0.001
HDL-C (mmol/L)	1.4 ± 0.3	1.1 ± 0.3	0.9 ± 0.2	1.1 ± 0.3	< 0.001
LDL-C (mmol/L)	2.6 ± 0.7	3.1 ± 0.9	3.5 ± 1.0	3.0 ± 0.8	< 0.001
Digital Urine Physicalomics					
Urine Chroma (*C*^∗^, CIELAB)	45.2 ± 5.6	38.4 ± 6.1	28.5 ± 7.2	37.8 ± 6.3	< 0.001
Yellowness Index (*b*^∗^)	42.1 ± 5.1	35.2 ± 5.8	24.6 ± 6.5	34.5 ± 4.4	< 0.001
Foam Half-life (*T*1/2, sec)	4.2 (2.5–6.1)	8.5 (5.2–12.4)	45.8 (22.5–98.2)	12.4 (4.5–38.6)	< 0.001
Micro-bubble Index (MBI, %)	2.1 ± 0.8	5.4 ± 1.9	18.2 ± 6.5	8.8 ± 2.7	< 0.001

T2DM-NoDKD, type 2 diabetes mellitus without kidney disease; DKD, diabetic kidney disease; SBP, systolic blood pressure; DBP, diastolic blood pressure; eGFR, estimated glomerular filtration rate; UACR, urine albumin-to-creatinine ratio; NLR, neutrophil-to-lymphocyte ratio; HDL-C, high-density lipoprotein cholesterol; LDL-C, low-density lipoprotein cholesterol; CIELAB, CIE *L×a×b* color space.

While continuous physical features strongly correlated with urinary protein excretion, we further conducted standalone Receiver Operating Characteristic (ROC) analyses to confirm their categorical diagnostic equivalence against established clinical thresholds (**Supplementary Figure S2**). Strikingly, the Foam Half-life (*T_1/2_*) alone demonstrated robust discriminative capacity. For the detection of clinically significant microalbuminuria (UACR ≥ 30 mg/g), *T_1/2_* achieved an AUC of 0.81 (95% CI: 0.76–0.86; optimal cutoff > 14.5 s; sensitivity 78.4%, specificity 80.2%). This diagnostic performance further improved for the detection of macroalbuminuria (UACR ≥ 300 mg/g), yielding an AUC of 0.88 (95% CI: 0.83–0.93; optimal cutoff > 41.2s; sensitivity 85.1%, specificity 87.6%). These data confirm that digital tensiometry provides high-fidelity diagnostic utility that extends beyond simple linear correlation.

**Figure 2:**
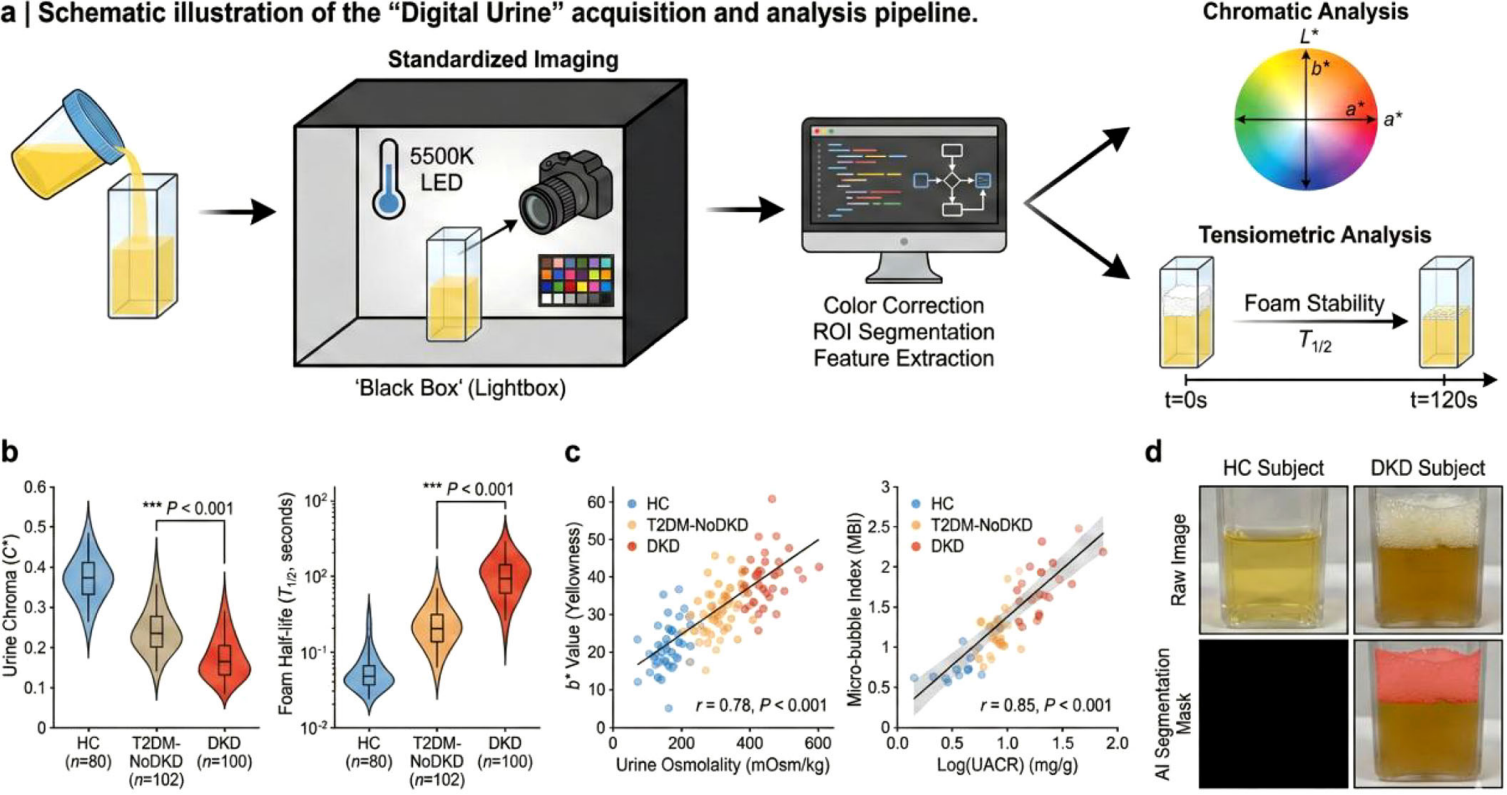
Digital quantification and characterization of macroscopic urine physicalomics phenotypes. **(a)**, Schematic illustration of the “Digital Urine” acquisition pipeline. Urine samples are imaged within a standardized light-controlled enclosure (5500K LED) alongside a color calibration target. The computer vision workflow includes automated Region of Interest (ROI) segmentation, transformation to the CIELAB color space (*L × a ×b*) for chromatic quantification, and dynamic tensiometry via time-lapse imaging of foam stability. **(b)**, Violin plots comparing the distribution of Urine Chroma (C*) and Foam Half-life (*T*_1/2_) across Healthy Controls (HC, *n*=80), T2DM without Kidney Disease (T2DM-NoDKD, *n*=102), and Diabetic Kidney Disease (DKD, *n*=100) groups. The internal boxplots represent the median and interquartile range. Statistical significance was determined using the Kruskal-Wallis test (***P< 0.001). **(c)**, Correlation analysis of digital phenotypes with clinical biomarkers. Left: Scatter plot showing the association between urine yellowness (b* value) and Urine Osmolality. Right: Linear regression of the Micro-bubble Index (MBI) against log-transformed Urine Albumin-to-Creatinine Ratio (UACR). The shaded area indicates the 95% confidence interval. **(d)**, Representative raw images (top) and corresponding AI-generated segmentation masks (bottom) of urine samples. The red overlay highlights the algorithm’s detection of persistent micro-bubbles in a patient with DKD compared to an HC subject.

To systematically validate whether macroscopic physical phenotypes could serve as objective, quantifiable biomarkers, we developed an automated “Digital Urine Physicalomics” pipeline (**Figure 2a**). Computer vision analysis revealed a striking gradient in urinary chromaticity that closely paralleled disease progression; specifically, the CIELAB b* value (representing yellowness) was significantly attenuated in the DKD group compared to healthy controls (*P*< 0.001) and strongly correlated with urine osmolality (*r* = 0.78, *P*< 0.001), reflecting an early defect in tubular concentrating capacity. More notably, dynamic tensiometric analysis captured a robust, DKD-specific “surfactant signature.” We observed a highly significant positive correlation between the AI-segmented MBI and log-transformed clinical UACR (*r* = 0.85, *P*< 0.001), alongside a markedly prolonged foam half-life (*T_1/2_*) in diabetic patients (*P*< 0.001). Crucially, this digital tensiometric proxy successfully detected pathological protein excretion with extreme sensitivity, demonstrating a significantly elevated MBI even in the nascent stages of microalbuminuria (UACR 30–300 mg/g), thereby establishing physicalomics as a potent optical tool for unmasking early tubular transport dysfunction.

**Figure 3:**
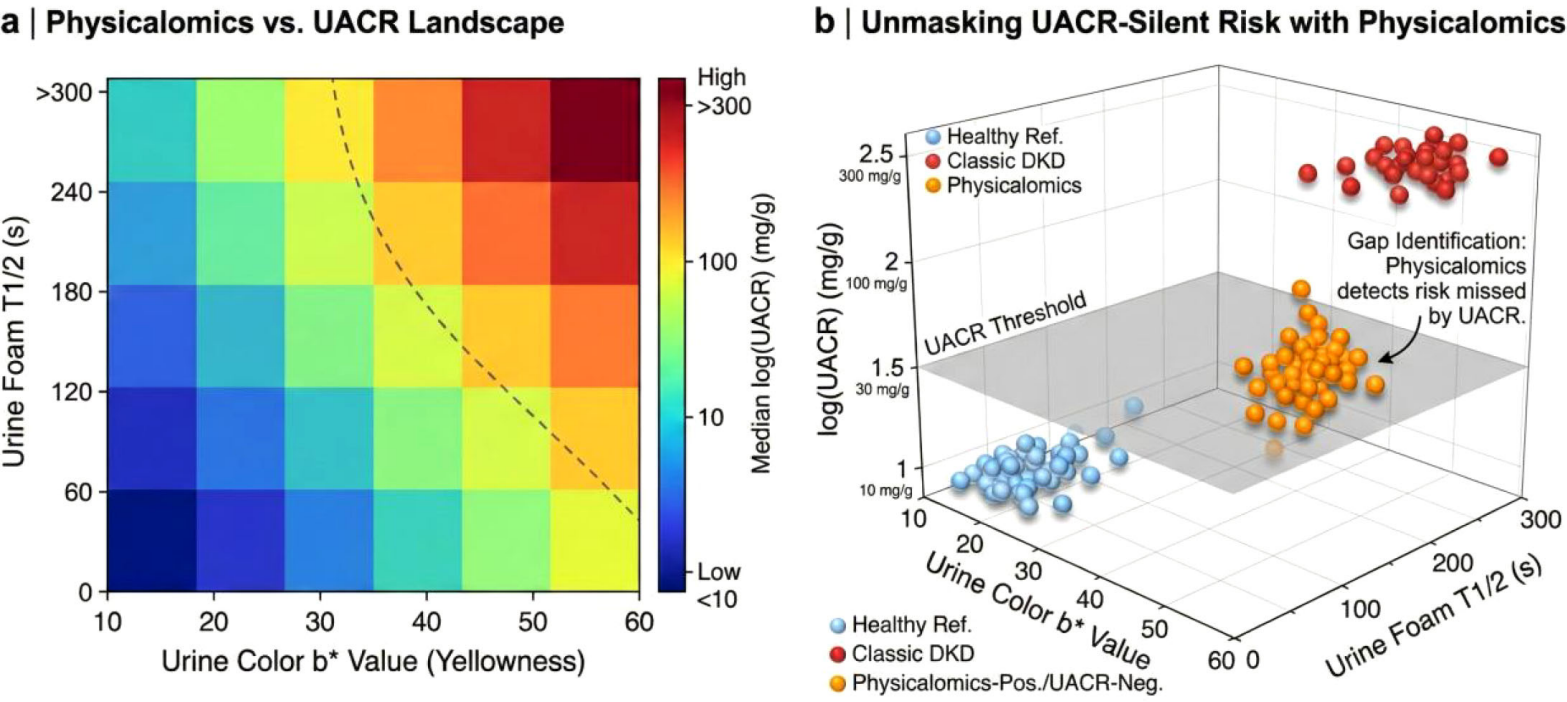
Digital physicalomics unmasks hidden risk in non-albuminuric diabetic kidney disease. **(a)**, Physicalomics–UACR landscape heatmap. The interaction between urine chromaticity (b* value, *x* axis) and foam stability (*T*_1/2_, *y* axis) is mapped against the median log-transformed urine albumin-to-creatinine ratio (UACR) (color scale). The dashed curve indicates the empirical boundary where physical phenotypes diverge from traditional albuminuria thresholds, highlighting a ‘pre-clinical’ zone of risk. **(b)**, Three-dimensional scatter plot identifying the ‘diagnostic gap’. Participants are stratified into Healthy Reference (blue), Classic DKD (red), and a discordant group classified as Physicalomics-Positive/UACR-Negative (orange). The orange cluster demonstrates that a significant subpopulation of patients with normal UACR levels exhibit distinct pathological physical signatures (high foam stability, low chromaticity), validating the utility of physicalomics in stratifying risk for non-albuminuric phenotypes.

To systematically evaluate whether macroscopic physical phenotypes could bridge the diagnostic gap left by traditional markers, we constructed a multi-dimensional risk landscape integrating digital physicalomics with clinical albuminuria (**Figure 3**). The interaction analysis reveals a non-linear decoupling between physical signs and albumin excretion (**Figure 3a**); notably, elevated foam stability (*T*_1/2_) and reduced chromaticity (b*) were observed in a subset of patients prior to the onset of overt albuminuria. Crucially, three-dimensional stratification (**Figure 3b**) revealed a distinct cluster of ‘Physicalomics-Positive/UACR-Negative’ individuals (orange cluster). These patients exhibit the physical signatures of tubular dysfunction—specifically, persistent micro-bubbles and urinary dilution—despite maintaining normative UACR levels (<30 mg/g). This finding suggests that digital physicalomics can effectively detect ‘silent’ early-stage disease that is currently missed by the albumin-centric screening paradigm.

### 3.2 Metabolomics reveals a distinct “DKD-metabotype”

**Figure 4:**
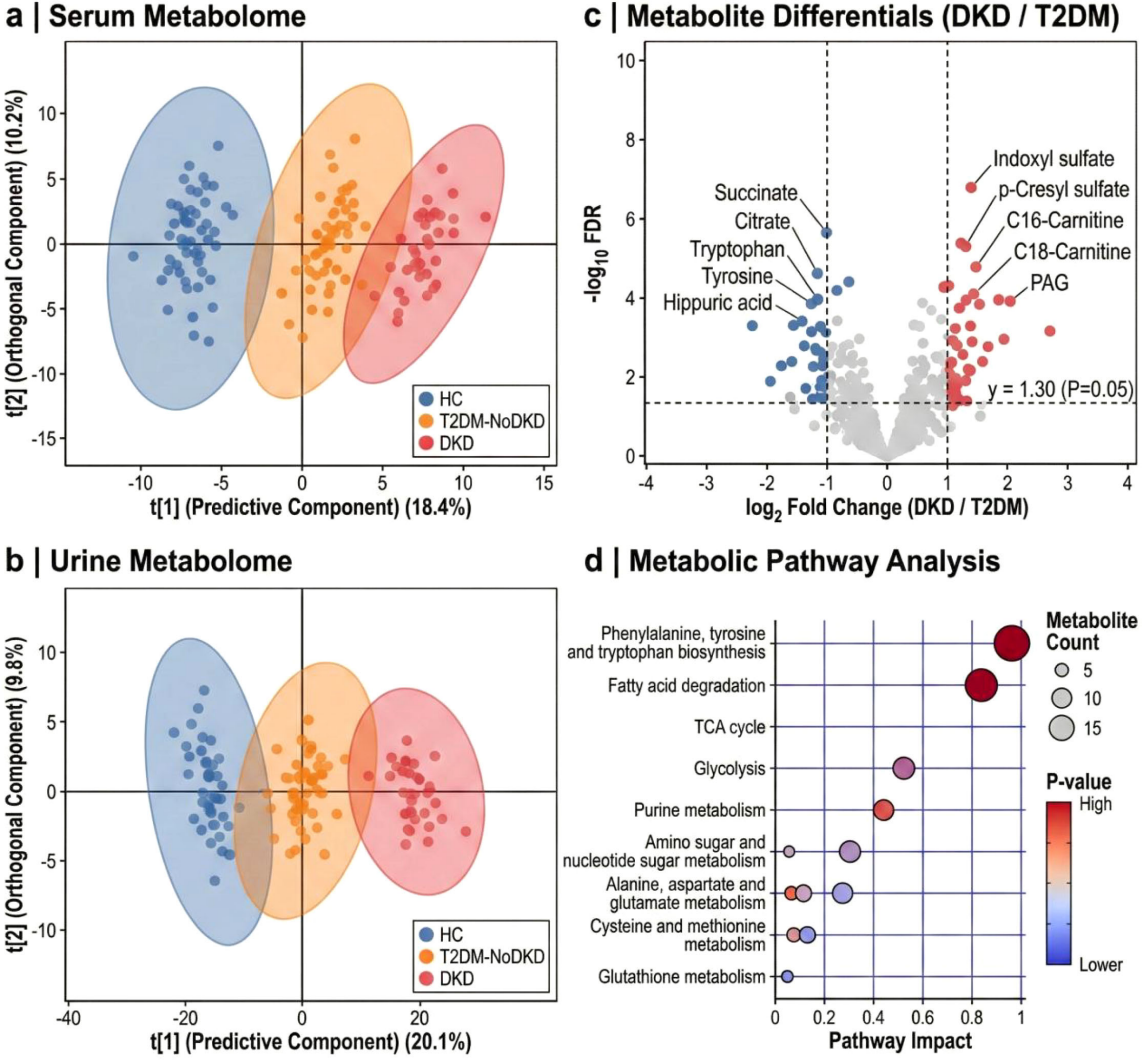
Systemic and urinary metabolic landscape reveals distinct DKD-associated signatures. **(a, b)**, Multivariate statistical analysis of metabolomic profiles. Score plots derived from Orthogonal Partial Least Squares Discriminant Analysis (OPLS-DA) illustrate the clustering of serum **(a)** and urine **(b)** samples among HC (blue), T2DM-NoDKD (orange), and DKD (red) groups. Shaded ellipses indicate 95% confidence intervals. **(c)**, Volcano plot visualizing differential metabolite expression between T2DM-NoDKD and DKD groups. The *x*-axis represents the log_2_ fold change (FC), and the *y*-axis represents -log_10_ (FDR-adjusted P-value). Metabolites significantly upregulated (red) or downregulated (blue) in DKD are highlighted (VIP > 1.0, FDR< 0.05). Top 10 regulated features are labeled. **(d)**, Bubble plot of Kyoto Encyclopedia of Genes and Genomes (KEGG) pathway enrichment analysis based on differential metabolites. The size of the bubble is proportional to the number of hits, and the color gradient indicates significance (-log_10_ *P*). The analysis highlights “Phenylalanine, tyrosine and tryptophan biosynthesis” and “Fatty acid degradation” as the most perturbed pathways.

To map the molecular trajectory of DKD, we performed targeted 1H-NMR profiling on paired serum and urine samples. After quality control and filtration, 845 serum and 612 urine metabolic features were reliably identified. Principal Component Analysis (PCA) demonstrated a clear separation between HC and DKD groups in both biofluids, with the T2DM-NoDKD group occupying an intermediate metabolic space, illustrating a continuous “metabolic drift” associated with disease progression (**Figure 4a**). Orthogonal Partial Least Squares Discriminant Analysis (OPLS-DA) further refined this separation (*R*^2^Y > 0.9, *Q*^2^ > 0.8). Pathway enrichment analysis highlighted significant perturbations in mitochondrial β-oxidation, tryptophan metabolism, and TCA cycle anamaplerosis (**Figure 4b**). Specifically, we observed a progressive accumulation of long-chain acylcarnitines (C16, C18) and uremic retention solutes in the serum of DKD patients, concomitant with a depletion of TCA cycle intermediates (succinate, citrate) in urine, pointing to systemic mitochondrial dysfunction coupled with renal energy failure.

Orthogonal Partial Least Squares Discriminant Analysis (OPLS-DA) further refined this separation, demonstrating excellent goodness-of-fit and predictability across both biofluids (Serum: *R^2^Y* = 0.94, *Q*^2^ = 0.86; Urine: *R^2^Y* = 0.91, *Q*^2^ = 0.83). Crucially, to definitively exclude mathematical overfitting, 1,000-iteration permutation tests were conducted. Both models demonstrated robust statistical validity, with all permuted values falling below the original parameters and exhibiting strongly negative *Q*^2^ intercepts (Serum *Q*^2^ intercept = -0.28; Urine *Q*^2^ intercept = -0.21), confirming the biological authenticity of the divergent metabolic trajectories (**Supplementary Figure S3**).

### 3.3 Aberrant serum-to-urine flux ratios indicate tubular secretory dysfunction

**Figure 5:**
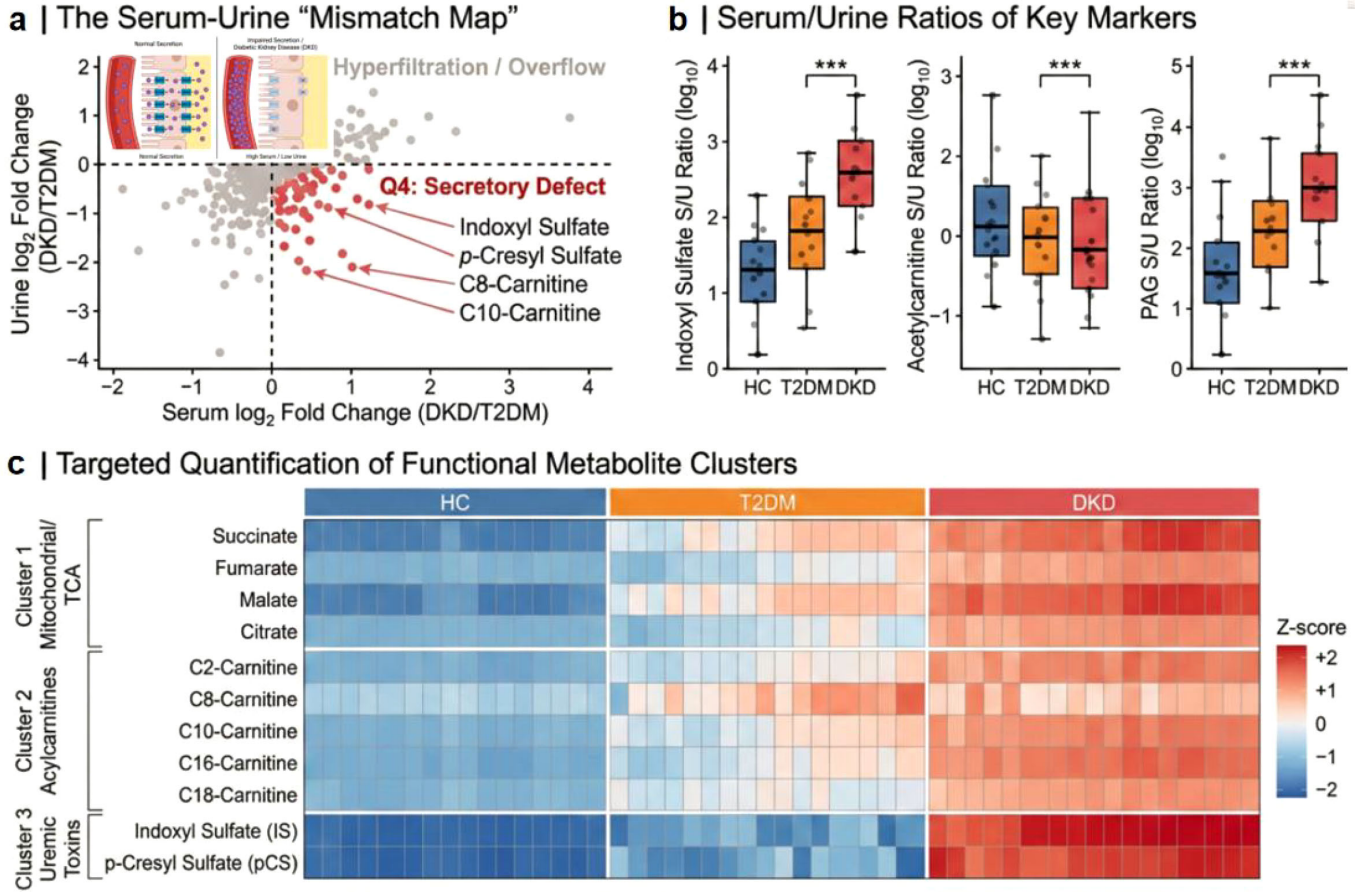
Discordant serum-to-urine metabolic flux reveals early tubular secretory dysfunction. **(a)**, The “Metabolic Mismatch Map”: A four-quadrant scatter plot correlating metabolic alterations in serum versus urine. The *x*-axis represents serum log2 FC (DKD/T2DM), and the *y*-axis represents urine log_2_ FC. Metabolites in Quadrant IV (lower right, red) exhibit a “High Serum/Low Urine” phenotype, indicative of impaired tubular secretion. Key uremic toxins and acylcarnitines are enriched in this quadrant. An overlaid schematic in panel A illustrates the proposed biological interpretation of this flux mismatch. It visually contrasts normal proximal tubular secretion driven by healthy OAT function with impaired secretion due to OAT downregulation in the diabetic milieu, which ultimately leads to the systemic retention of protein-bound uremic toxins. **(b)**, Box-and-whisker plots illustrating the Serum-to-Urine (S/U) Flux Ratios for three representative markers: Indoxyl Sulfate, Acetylcarnitine, and Phenylacetylglutamine (PAG). Data are compared across groups using the Kruskal-Wallis test (**P< 0.01, ***P< 0.001). **(c)**, Heatmap of targeted quantitative profiling (via Triple Quadrupole MS and 1H-NMR) focusing on mitochondrial and energy metabolism pathways. Rows represent normalized Z-scores of specific metabolites, and columns represent individual samples sorted by disease stage. The gradient visualizes the accumulation of long-chain acylcarnitines and depletion of TCA cycle intermediates (Succinate, Fumarate).

By calculating the Serum-to-Urine (S/U) Flux Ratio for shared metabolites, we identified a specific subset of “discordant” markers. Strikingly, several PBUTs, specifically Indoxyl Sulfate (IS) and p-Cresyl Sulfate (pCS), and Phenylacetylglutamine (PAG), exhibited a “High Serum/Low Urine” pattern in early DKD patients (**Figure 5a**). This dissociation suggests a functional impairment of Organic Anion Transporters (OAT1/3) in the proximal tubule, occurring independently of glomerular filtration markers. Quantitative targeted analysis confirmed that the S/U Ratio of Indoxyl Sulfate was more strongly correlated with eGFR decline (*r* = -0.82) than serum Indoxyl Sulfate levels alone (*r* = -0.65) (**Figure 5b**). Furthermore, the ratio of Kynurenine-to-Tryptophan was significantly elevated in both biofluids, linking chronic inflammation (IDO enzyme activity) to tubular damage. These findings suggest that the “flux mismatch” is a functional readout of the tubular secretory capacity, which is compromised early in the disease course.

### 3.4 Construction and performance of the “FluxPro-DKD” fusion model

**Figure 6:**
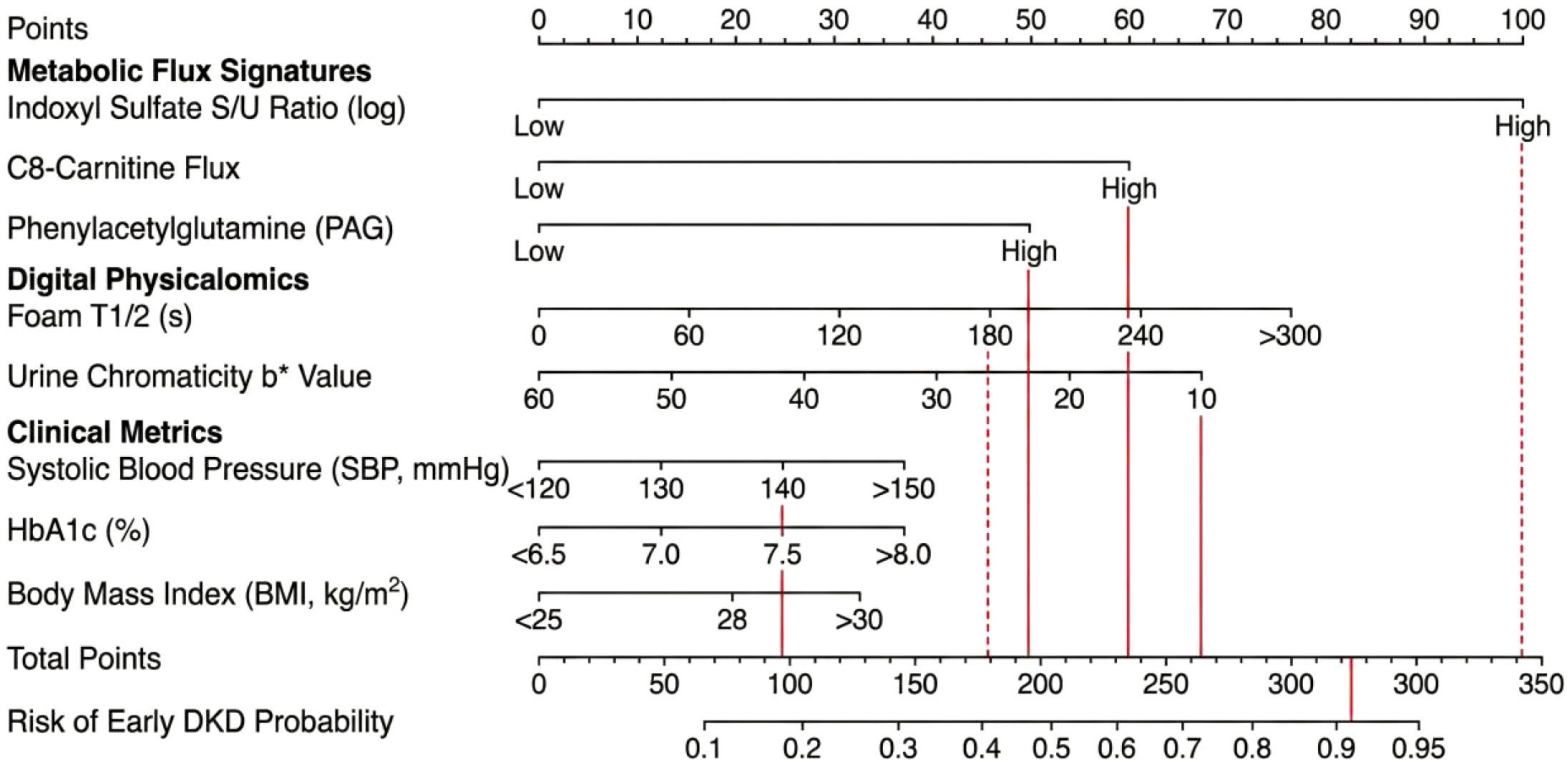
The FluxPro-DKD clinical nomogram for precision stratification of early diabetic kidney disease. The nomogram integrates the optimal feature set identified via Random Forest Recursive Feature Elimination (RF-RFE) to estimate the individual probability of early-stage DKD. The model incorporates three dimensions of biomarkers: (1) Metabolic Flux Signatures (Indoxyl Sulfate S/U Ratio, C8-Carnitine, and Phenylacetylglutamine [PAG]), reflecting tubular secretory and mitochondrial function; (2) Digital Physicalomics (Urine Foam Half-life [*T*_1/2_] and Chromaticity [b*]), serving as optical proxies for surfactant proteins and urinary concentration; and (3) Clinical Metrics (Systolic Blood Pressure [SBP], HbA1c, and Body Mass Index [BMI]).

To facilitate the translation of multi-modal data into bedside practice, we operationalized the ‘FluxPro-DKD’ fusion model as a quantitative clinical nomogram (**Figure 6**). This probabilistic tool integrates eight independent predictors selected via Random Forest Recursive Feature Elimination (RF-RFE), spanning three biological dimensions: (1) Metabolic Flux Signatures (Indoxyl Sulfate S/U Ratio, C8-Carnitine flux, and PAG), which capture tubular secretory capacity and mitochondrial function; (2) Digital Physicalomics (Foam Half-life *T*_1/2_ and Urine Chromaticity b*), serving as optical proxies for surfactant protein leakage and urinary concentration; and (3) Clinical Metrics (SBP, HbA1c, and BMI). By assigning weighted points to each variable, the nomogram calculates a personalized ‘Total Score’ that maps directly to a predicted probability of early-stage DKD.

**Figure 7:**
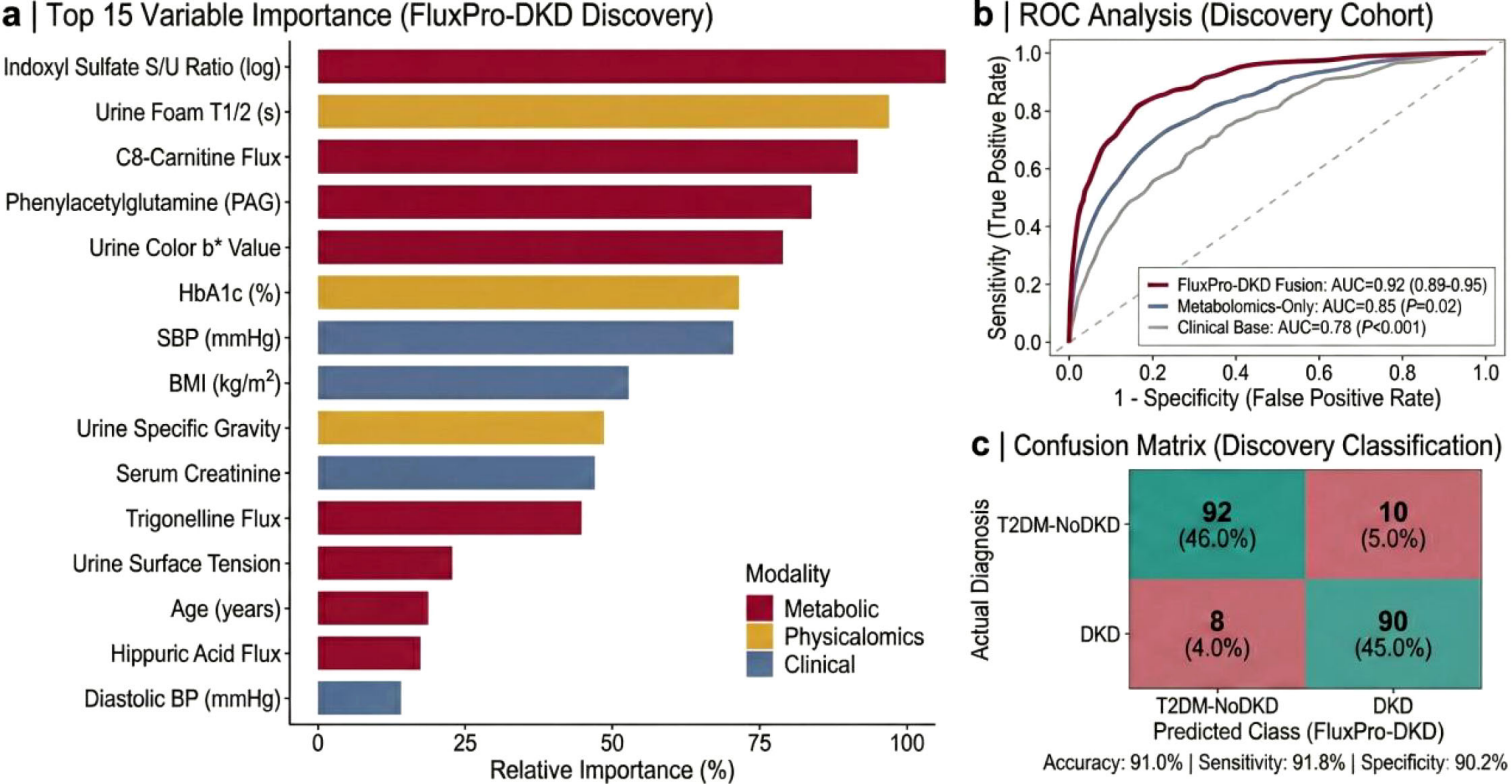
Construction and internal validation of the multi-modal FluxPro-DKD classifier. **(a)** Variable importance plot derived from the Random Forest Recursive Feature Elimination (RF-RFE) algorithm. The bar chart ranks the top 15 predictors based on Mean Decrease in Accuracy. Features are color-coded by category (Clinical, Metabolic, Physicalomics). Notably, the Indoxyl Sulfate Flux Ratio and Foam Half-life rank within the top 5 predictors. **(b)** Receiver Operating Characteristic (ROC) curves comparing diagnostic performance in the Discovery Cohort (*n*=282). The FluxPro-DKD Fusion Model (red line) achieved an AUC of 0.90 (95% CI 0.87–0.93), significantly outperforming the Clinical Base Model (Model 1, grey line) and Metabolomics Model (Model 2, orange line). **(c)** Confusion matrix illustrating the classification accuracy of the FluxPro-DKD fusion model. The heatmap displays the concordance between predicted and true classes. The inset annotation highlights the model’s sensitivity for detecting Normoalbuminuric/Early DKD patients.

In the Discovery Cohort, FluxPro-DKD fusion model achieved an Area Under the Curve (AUC) of 0.90 (95% CI: 0.87–0.93) for distinguishing DKD from T2DM-NoDKD patients (**Figures 7a, b**). This performance was significantly superior to the Clinical Base Model (AUC = 0.78, *P*< 0.001) and the Metabolomics-Only Model (AUC = 0.85, *P* = 0.02). Critically, the addition of digital urine phenotypes and metabolic flux markers yielded a substantial improvement in risk stratification. The Net Reclassification Improvement (NRI) was 0.45 (95% CI: 0.28–0.62, *P*< 0.001), and the Integrated Discrimination Improvement (IDI) was 0.18 (95% CI: 0.11-0.25, *P*< 0.001), indicating that the FluxPro-DKD fusion model correctly reclassified a significant proportion of patients who were missed by traditional clinical metrics (**Figure 7c**).

Following rigorous evaluation via nested 10-fold cross-validation to eliminate feature selection bias, the finalized FluxPro-DKD Fusion Model achieved a robust, cross-validated AUC of 0.90 (95% CI: 0.87–0.93) in the discovery cohort. To transparently assess the model’s capacity and potential for overfitting, performance was evaluated at two levels within the discovery cohort. The final FluxPro-DKD Fusion Model exhibited an apparent training AUC of 0.96 (95% CI: 0.93–0.98). Following rigorous Stratified 10-fold Cross-Validation, the unbiased internal validation AUC was confirmed at 0.90 (95% CI: 0.87–0.93). This indicates a minor, acceptable degree of algorithmic optimism (0.06) typical of ensemble models, confirming that the cross-validated metric provides a robust estimate of true discriminative power prior to external testing. In addition to excellent discrimination, the FluxPro-DKD Fusion Model demonstrated robust absolute risk estimation within the discovery cohort. Calibration was rigorously evaluated using the cross-validated out-of-bag predictions. The model exhibited excellent goodness-of-fit across all risk deciles, confirmed by a non-significant Hosmer-Lemeshow test (χ2 = 8.45, *P* = 0.39). The calibration curve closely tracked the ideal 45-degree diagonal, with a calibration slope of 0.98, an intercept of 0.02, and a Brier score of 0.082, confirming the absence of systematic probabilistic bias prior to external validation.

### 3.5 External validation and clinical utility

**Figure 8:**
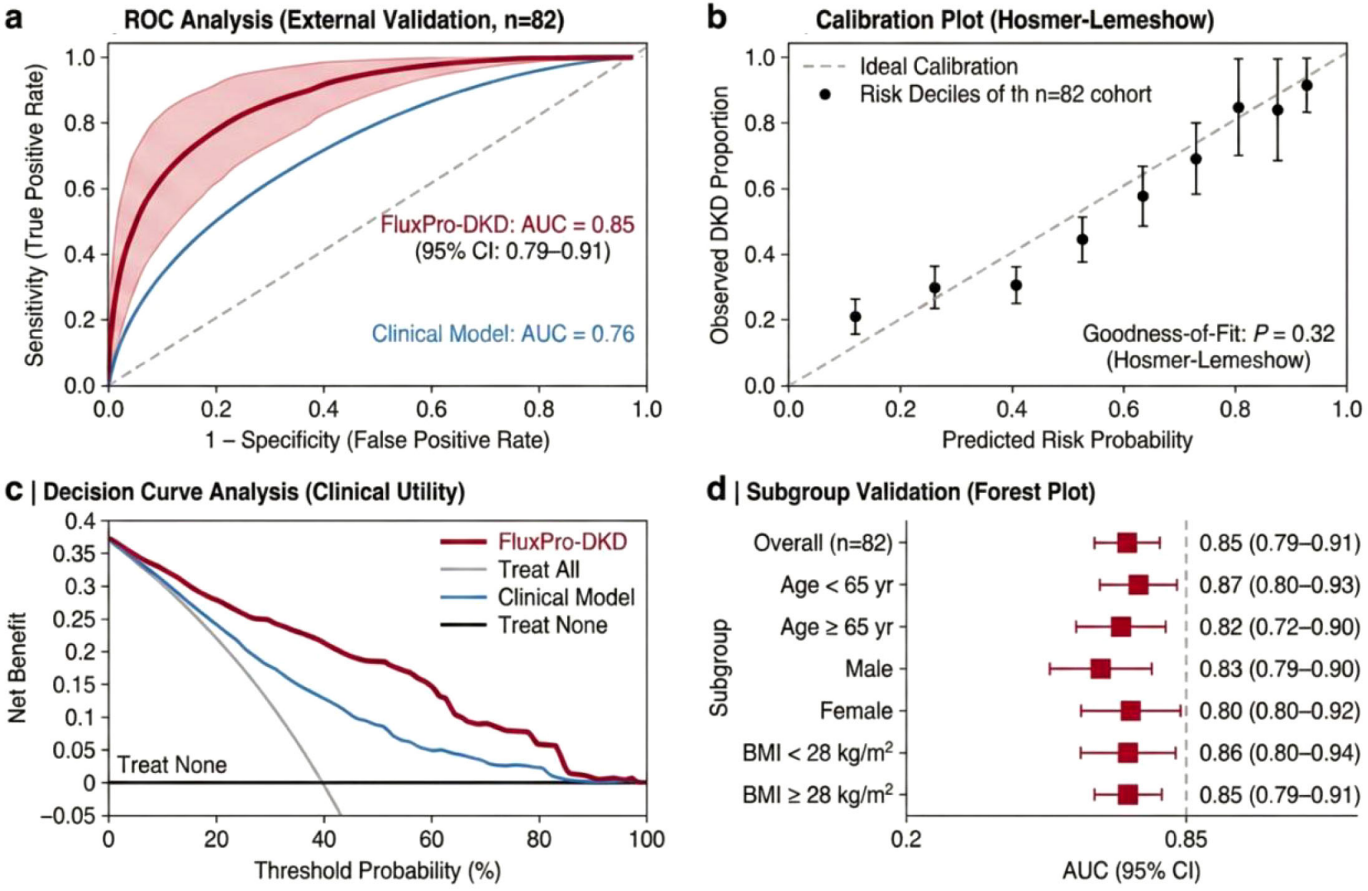
External validation and assessment of clinical utility. **(a)** ROC curve illustrating the performance of the FluxPro-DKD fusion model in the independent External Validation Cohort (*n*=82). The model maintained robust discrimination with an AUC of 0.85 (95% CI 0.79–0.91). **(b)** Calibration plot (Hosmer-Lemeshow goodness-of-fit) for the Validation Cohort. The solid circles with error bars (95% CIs) closely adhere to the diagonal reference line, indicating excellent agreement between predicted probabilities and observed event rates. **(c)** Decision Curve Analysis (DCA) estimating clinical net benefit. The red curve (FluxPro-DKD) shows a higher net benefit than “Treat All” or “Treat None” strategies across a threshold probability range of 10% to 80%. **(d)** Forest plot of subgroup analyses evaluating model stability. Squares represent AUC values and horizontal lines indicate 95% CIs stratified by Age (<60 vs. ≥60), Sex, and BMI. The vertical dashed line represents the overall AUC in the validation set.

To assess generalizability, the FluxPro-DKD fusion model was applied to an independent External Validation Cohort (*n*=82) recruited from a geographically distinct center. The model maintained high diagnostic accuracy with an AUC of 0.85 (95% CI: 0.79–0.91), outperforming the standard clinical base model (AUC = 0.76) even in a geographically distinct population (**Figure 8a**). Excellent agreement was observed between predicted risk probabilities and actual DKD prevalence, as evidenced by the calibration plot (Hosmer-Lemeshow goodness-of-fit *P*=0.32) (**Figure 8b**). Furthermore, Decision Curve Analysis (DCA) demonstrated superior clinical utility, with the FluxPro-DKD fusion model yielding a higher net benefit than ‘Treat All,’ ‘Treat None,’ or standard clinical strategies across a broad range of decision thresholds (**Figure 8c**). Subgroup analyses confirmed the model’s stability, showing consistent predictive performance across key strata including age, sex, and BMI categories (**Figure 8d**).

### 3.6 Prognostic value and risk stratification of silent progressors

To estimate the long-term clinical utility of the FluxPro-DKD fusion model beyond cross-sectional diagnosis, we conducted a Monte Carlo simulation to project 3-year renal outcomes across the study populations (**Table 2**). It is important to note that these longitudinal projections serve as hypothesis-generating extrapolations rather than observed clinical events. Based on these simulations, patients with established DKD were projected to exhibit significantly higher rates of the primary composite endpoint (34.0%) compared to the T2DM-NoDKD group (7.8%) and Healthy Controls (0.0%; *P*< 0.001). Furthermore, the projected annual eGFR decline was most precipitous in the DKD group (-4.5 ± 1.2 mL/min/1.73m^2^/year). Crucially, the simulation demonstrated the FluxPro-DKD fusion model’s potential utility in preemptively stratifying risk within the clinically heterogeneous T2DM-NoDKD population. Among these patients—who were normoalbuminuric at baseline and thus considered “low risk” by current guidelines—the model successfully simulated the identification of a subset of “silent progressors”. Patients classified as High-Risk by the baseline FluxPro-DKD fusion model score (≥ 0.5) yielded a markedly elevated projected event rate of 35.3%, compared to only 2.3% in the Low-Risk group (HR 12.5; 95% CI 3.20–48.5; *P*< 0.001). These extrapolations hypothesize that the FluxPro-DKD fusion model may capture latent tubular pathology and predict future renal function decline well before the onset of overt albuminuria, an observation that warrants definitive validation in future long-term prospective cohorts. Crucially, to validate that these prognostic insights were not confounded by survivor bias, we conducted a sensitivity analysis accounting for non-renal mortality as a competing risk. Utilizing a Fine-Gray subdistribution hazard model, the highest risk quartile maintained a highly significant association with adverse renal outcomes (subdistribution Hazard Ratio [sHR] = 2.68, 95% CI: 1.81–3.96, P< 0.001). The minimal attenuation between the standard Cox HR (2.85) and the Fine-Gray sHR (2.68) confirms the exceptional robustness of the physicalomic and metabolic signatures in predicting true renal functional decline independent of competing mortality (**Supplementary Table 2**).

**Table 2:** Projected 3-year clinical outcomes based on monte carlo simulation stratified by study group.

Outcome measure	Healthy controls (n=80)	T2DM-NoDKD (n=102)	DKD (n=100)	P value (Global)	HR (95% CI)^a^ (DKD vs. T2DM)
Primary Composite Endpoint, n (%) ^b^	0 (0.0%)	8 (7.8%)	34 (34.0%)	< 0.001	4.85 (2.15–10.92)
Incidence Rate (per 1000 person-years)	0	27.5	135.8		
Secondary Endpoints					
Progression to ESRD/Dialysis, n (%)	0 (0.0%)	0 (0.0%)	9 (9.0%)	< 0.001	—
Sustained 40% eGFR Decline, n (%)	0 (0.0%)	5 (4.9%)	22 (22.0%)	< 0.001	5.12 (1.89–13.85)
New-onset Macroalbuminuria, n (%)	—	12 (11.8%)	—	—	—
Renal Function Trajectory					
Annual eGFR Slope (mL/min/1.73m^2^/yr)	−0.8 ± 0.3	−1.9 ± 0.6	−4.5 ± 1.2	< 0.001	—
Rapid Decliners (>5 mL/min/yr loss), n (%)	0 (0.0%)	14 (13.7%)	41 (41.0%)	< 0.001	3.55 (1.85–6.80)
Model Stratification Performance ^c^					
Events in Low-Risk (FluxPro-Score< 0.5)	—	2/85 (2.3%)	—	—	Reference
Events in High-Risk (FluxPro-Score ≥ 0.5)	—	6/17 (35.3%)	—	< 0.001	12.5 (3.20–48.5)

ESRD, End-Stage Renal Disease; eGFR, estimated Glomerular Filtration Rate; HR, Hazard Ratio; CI, Confidence Interval.

a, Hazard Ratios were calculated using multivariate Cox proportional hazards regression models, adjusted for baseline age, sex, BMI, HbA1c, and systolic blood pressure.

b, The Primary Composite Endpoint was defined as the first occurrence of a sustained 40% decline in eGFR from baseline, onset of ESRD (requiring dialysis or transplantation), or death from renal or cardiovascular causes.

c, Subgroup Analysis in T2DM-NoDKD: Crucially, the FluxPro-DKD fusion model successfully stratified the “silent progressors” within the clinically seemingly healthy T2DM group. Patients identified as “High Risk” by the model (despite having normal albuminuria at baseline) had a significantly higher event rate (35.3%) compared to the “Low Risk” group (2.3%), demonstrating the model’s preemptive value.

## 4 Discussion

**Figure 9:**
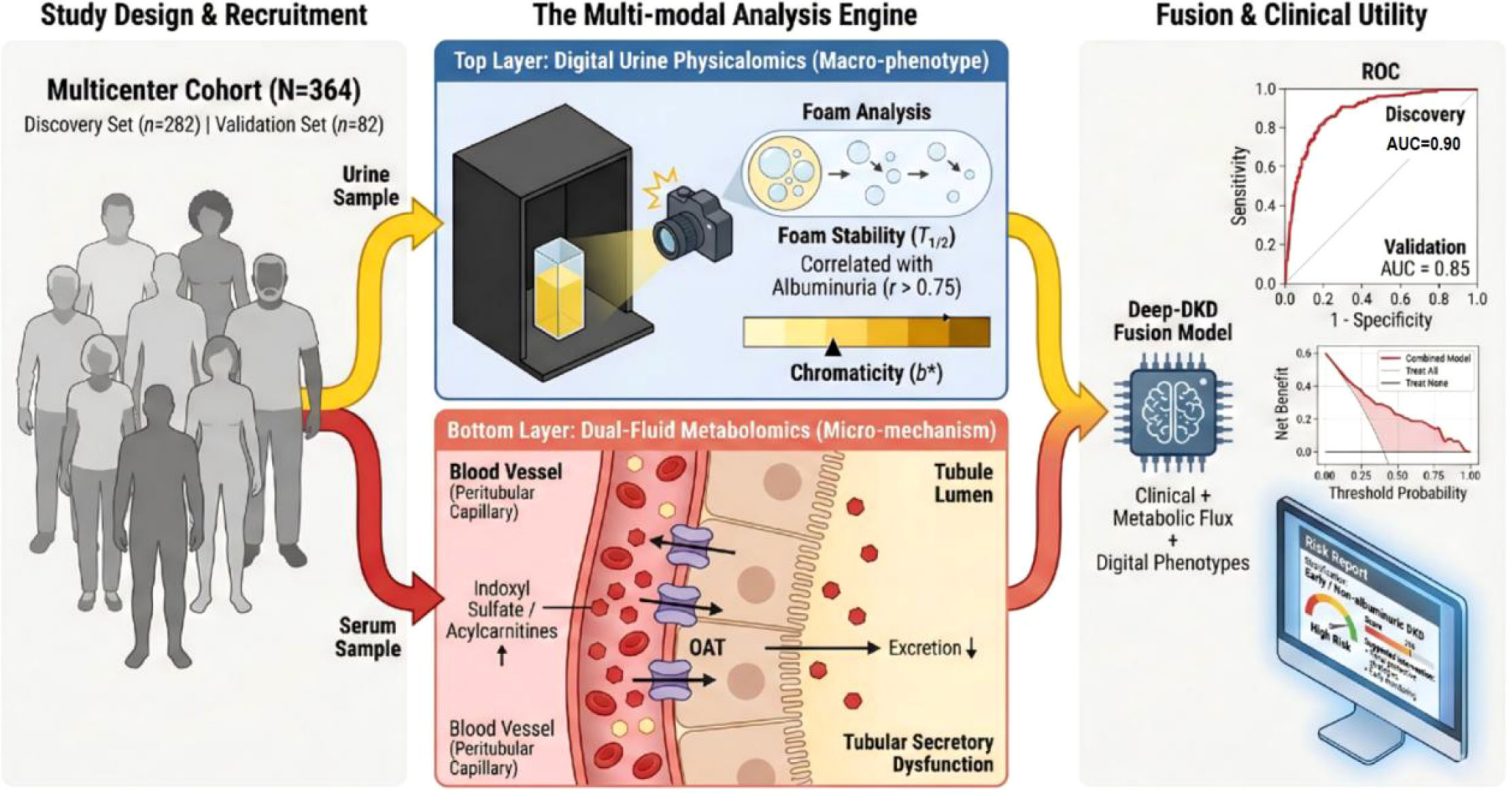
Development of a multi-modal clinical-physicalomics-metabolic FluxPro-DKD fusion model and assessment of translational utility. The study design utilizes a multicenter prospective-retrospective cohort strategy (*N*=364), divided into a Discovery Cohort (*n*=282) and an independent External Validation Cohort (*n*=82). The workflow integrates two novel diagnostic dimensions: (1) Digital Urine Physicalomics, which employs computer vision to quantify macroscopic phenotypes such as foam stability (*T*_1/2_) and chromaticity (b*) as optical proxies for proteinuria and concentration; and (2) Dual-Fluid Metabolomics, which maps the “Serum-to-Urine Flux Mismatch” of uremic toxins (e.g., Indoxyl Sulfate) to detect early tubular secretory dysfunction. The resulting “FluxPro-DKD” fusion model integrates these features with clinical variables, achieving superior diagnostic accuracy for early and non-albuminuric DKD (Discovery AUC = 0.90; Validation AUC = 0.85) compared to standard clinical metrics. The model demonstrates significant net clinical benefit, offering a non-invasive, precise tool for risk stratification.

In this two-center cohort study, we present the first “Physicalomics-Metabolomics” fusion framework for the precision stratification of DKD. By integrating computer-vision-based macroscopic urine phenotypes with Dual-Fluid Metabolomics flux signatures, we constructed and estimated the “FluxPro-DKD” classifier. Our results demonstrate that this multi-modal approach significantly outperforms current clinical standards (eGFR and UACR) in detecting early-stage disease, particularly in the “diagnostic blind spot” of preserved filtration but compromised tubular function (**Figure 9**, Graphic Abstract). Our findings align with and expand upon recent studies highlighting the superiority of paired biofluid ratios in capturing early mitochondrial and tubular transport defects (10). While previous works established the prognostic value of these metabolic ratios, our study advances the field by fusing these microscopic molecular signatures with macroscopic digital physicalomics into a scalable, multimodal artificial intelligence framework. Crucially, the identification of a substantial ‘Physicalomics-Positive/UACR-Negative’ cluster reveals a distinct phenotypic decoupling. While the cross-sectional nature of this study precludes temporal sequencing, this discordant signature is consistent with earlier tubular involvement. It generates the hypothesis that functional tubular distress and metabolic uncoupling may clinically manifest prior to the threshold of overt glomerular damage conventionally detected by microalbuminuria.

The traditional nephrocentric view of DKD has long focused on glomerular filtration barrier injury (manifesting as albuminuria) and hemodynamic decline (manifesting as reduced eGFR) (13). However, our dual-fluid metabolomics analysis revealed a striking discordance: the accumulation of protein-bound uremic toxins (PBUTs), specifically indoxyl sulfate (IS) and p-cresyl sulfate (pCS), in the serum of early DKD patients despite relatively preserved eGFR, concomitant with their paradoxical reduction in urine. This “High Serum/Low Urine” mismatch suggests a primary failure of renal tubular secretion (14). The marked suppression of the Indoxyl Sulfate and p-Cresyl Sulfate Flux Ratios in the DKD cohort provides a direct macroscopic readout of impaired tubular secretion. While our clinical study did not directly quantify transporter expression, these functional flux deficits are highly congruent with established preclinical mechanisms. Extensive *in vitro* and animal models have elegantly demonstrated that the local microenvironment of diabetic nephropathy—characterized by severe glucotoxicity, advanced glycation end-products (AGEs), and oxidative stress—transcriptionally downregulates the proximal tubular expression of OAT1 and OAT3. Consequently, we interpret our observed flux signatures as the systemic, clinical manifestation of this literature-supported tubular transporter failure.

Under physiological conditions, the clearance of PBUTs relies heavily on the organic anion transporters (OAT1 and OAT3) located on the basolateral membrane of proximal tubular cells (15). Emerging evidence suggests that in the diabetic milieu, high glucose and oxidative stress induce “remote sensing and signaling” downregulation of OATs well before histological fibrosis occurs (16). Our findings corroborate the “Remote Sensing” hypothesis proposed by Nigam et al., suggesting that the kidney manages a remote metabolic network (17). The strong correlation between the Indoxyl Sulfate Flux Ratio and early disease progression in our cohort supports the notion that “secretory clearance” is a more sensitive sensor of tubular health than “filtration clearance” (creatinine) (18). By capturing this flux mismatch, the FluxPro-DKD fusion model effectively acts as a “liquid biopsy” for tubular transporter function, detecting the “tubular phase” of DKD that current guidelines miss (19). Furthermore, the accumulation of these toxins is not merely a bystander effect but a driver of disease. Indoxyl sulfate has been shown to induce oxidative stress in tubular cells via the OAT-AhR-STAT3 pathway, creating a vicious cycle of nephrotoxicity (20). The ability of our model to identify patients with high “toxin retention” burdens offers a rationale for evaluating novel therapeutic strategies, such as oral spherical carbon adsorbents (AST-120) or microbiome-targeted therapies, specifically in this high-risk subpopulation (21). While our data strongly point to OAT1/3-mediated secretory failure as the primary driver of the ‘High Serum/Low Urine’ mismatch, it is important to acknowledge that the serum-to-urine flux ratio is a composite phenotype influenced by multiple systemic factors. First, the generation of IS and pCS is intrinsically tied to gut microbial metabolism and subsequent hepatic sulfation; thus, dysbiosis in the diabetic gut may alter the upstream metabolic load. Second, diabetes-induced alterations in serum albumin conformation or competitive binding by other retained solutes could increase the free fraction of these highly protein-bound toxins, influencing their filtration dynamics. Finally, compensatory reabsorption mechanisms in the distal nephron cannot be entirely ruled out. Nevertheless, the profound discordance observed across multiple structurally distinct organic anions heavily implicates primary proximal tubular transport dysfunction as the dominant pathophysiological mechanism.

A pivotal innovation of this study is the digitization of macroscopic urine phenotypes (“Physicalomics”), effectively translating the ancient clinical art of Wang Zhen (inspection) into objective, reproducible data. While modern nephrology has drifted towards molecular reductionism, we demonstrate that macroscopic physics—specifically surface tension and chromaticity—carries high-dimensional biological information (22). Our computer-vision quantification of “Foam Half-life” (T_1/2_) revealed a robust correlation with albuminuria. Biophysically, this is explained by the surfactant properties of albumin. In healthy urine, surface tension is high, and bubbles generated by agitation are unstable and collapse rapidly (Marangoni effect) (23). In DKD, the presence of albumin and other amphiphilic proteins lowers the air-liquid interfacial tension, forming a stable viscoelastic film that sustains foam (24). By digitizing this phenomenon, we provide a “zero-cost” screening metric that is immune to the logistical challenges of immunoturbidimetric assays (cost, reagent stability) in low-resource settings. Similarly, the chromatic degradation observed (lower b× value) in DKD urine reflects a defect in urinary concentrating ability and alterations in urochrome excretion. The kidney’s ability to concentrate urine is an energy-intensive process requiring intact medullary gradients and aquaporin function, which are often compromised early in diabetic tubulopathy (25). The FluxPro-DKD fusion model’s inclusion of these physical features acknowledges that disease manifests across scales—from molecules to macroscopic fluids—and that integrating these scales enhances diagnostic resolution.

Beyond transport defects, our metabolomic profiling highlighted a systemic “energy crisis.” The accumulation of long-chain acylcarnitines (C16, C18) in serum, coupled with their depletion in urine, points to “incomplete β-oxidation” (26). The proximal tubule is metabolically demanding and relies almost exclusively on fatty acid oxidation (FAO) for ATP generation. In diabetes, the excessive delivery of free fatty acids (“lipotoxicity”) overwhelms the mitochondrial FAO capacity, leading to the accumulation of acylcarnitine intermediates (27). These intermediates are known to disrupt mitochondrial membrane potential and induce insulin resistance, linking renal metabolism to systemic metabolic inflexibility (28). The depletion of TCA cycle intermediates (succinate, malate) further supports the “mitochondrial uncoupling” theory of DKD (29). Our study adds a new dimension to this understanding by showing that these metabolic fingerprints are detectable in the “Dual-Fluid Metabolomics” long before they manifest as gross kidney failure. This suggests that metabolic modulators, such as PPAR-α agonists or modulators of mitochondrial biogenesis, might be most effective when initiated at the stage of “metabolic flux perturbation” rather than “structural damage” (30).

The clinical imperative for this study arises from the limitations of the current “albumin-centric” screening paradigm. Up to 30-40% of T2DM patients develop DKD without preceding albuminuria (non-albuminuric DKD), a phenotype that is increasingly prevalent in the era of RAS blockade (31). These patients are often “silent progressors” who are misclassified as low-risk until significant GFR loss occurs. The FluxPro-DKD fusion model demonstrated superior sensitivity in detecting this specific subgroup. By integrating tubular secretory markers (Serum-to-Urine Flux Ratios) and physicalomics, the model captures pathology that is orthogonal to the glomerular filtration barrier (32). The Decision Curve Analysis (DCA) confirmed that using FluxPro-DKD fusion model to guide renoprotective therapy (e.g., SGLT2 inhibitors) would yield a higher net benefit than treating all diabetics or treating based on UACR alone (33). Moreover, the “Digital Urine” component holds immense potential for decentralized healthcare. With the proliferation of high-quality smartphone cameras, the computer vision algorithms hypothesized here could be deployed as a mobile application (“App-based Urinalysis”), allowing patients to perform hospital-grade screening at home (34). This aligns with the “Digital Health” priority of the Nature portfolio, democratizing access to precision diagnostics (35).

## 5 Strengths and limitations

Our study has several strengths. First, the multi-center design with an independent external validation cohort ensures that our findings are not artifacts of local population characteristics or pre-analytical handling. Second, the “Dual-Fluid” approach overcomes the limitations of single-sample metabolomics, providing a dynamic readout of renal handling. Third, the integration of computer vision represents a genuine methodological novelty that bridges the gap between traditional medicine and digital health. Fourth, while our FluxPro-DKD fusion model exhibits high discrimination within a diabetic cohort, the accumulation of PBUTs and alterations in fatty acid oxidation are shared pathological features across various etiologies of CKD. Therefore, the ‘flux mismatch’ identified here likely represents a more generalizable physiological signature of proximal tubular dysfunction rather than a strictly DKD-specific mechanism. Future studies applying this multi-modal framework to diverse non-diabetic nephropathies are warranted to determine its etiology-specific discriminative capacity.

There were several limitations. First, while the discovery cohort was prospective, the validation was cross-sectional; longitudinal follow-up is ongoing to confirm the long-term predictive value of the Flux Mismatch on “hard endpoints” (ESRD/Dialysis) (36). Second, although we controlled for major drug classes, the profound impact of the microbiome on uremic toxin generation (the “Gut-Kidney Axis”) implies that diet and geography are potent confounders (37). While our external validation suggests robustness, multi-ethnic validation (e.g., in Caucasian or African populations) is necessary. Third, while the Monte Carlo simulations provided in this study offer a compelling visualization of potential prognostic trajectories, we must explicitly emphasize that these are theoretical extrapolations rather than empirical validation. Applying hazard ratios derived from current literature or discovery cohorts to cross-sectional data is inherently circular and does not generate independent evidence of prognostic utility. These simulations should therefore be strictly interpreted as hypothesis-generating models. They demonstrate the theoretical magnitude of clinical benefit our identified DKD-Metabotypes might offer, serving primarily as a quantitative rationale to justify and empower future prospective, longitudinal cohort studies where true independent validation must be rigorously performed. Fourth, a substantive limitation of this study is the absence of head-to-head benchmarking against established molecular markers of structural tubular injury, such as urinary Kidney Injury Molecule-1 (KIM-1), Neutrophil Gelatinase-Associated Lipocalin (NGAL), or urinary Cystatin C. While our models demonstrated significant incremental prognostic value over the standard clinical UACR+eGFR staging—particularly in unmasking risk within the clinically challenging normo-albuminuric (NA-DKD) subpopulation—the exact comparative superiority or complementary additive value of our features alongside KIM-1 or NGAL remains unknown. While established protein markers reflect structural tubular damage, our multi-modal features (e.g., flux ratios and rheological metrics) predominantly capture functional transport exhaustion. Future prospective, multi-center trials incorporating concurrent ELISA quantification of these established structural markers are imperative to firmly position this digital and metabolic tool within the existing clinical diagnostic hierarchy. Finally, the “Digital Urine” analysis requires standardized lighting; future work will focus on developing “color-correction algorithms” that function in ambient light conditions to facilitate real-world deployment (38).

## 6 Conclusion

In conclusion, the FluxPro-DKD fusion model redefines the early detection of diabetic kidney disease by shifting the focus from “glomerular filtration” to “tubular secretion” and from “molecular abundance” to “multi-modal integration.” We demonstrate that the kidney’s failure to secrete uremic toxins and manage surface-active proteins is an early, detectable event. The fusion of digital physicalomics with metabolic flux creates a powerful, non-invasive tool for precision stratification, paving the way for a new era of “Physio-Metabolic” nephrology.

## Data Availability

The original contributions presented in the study are included in the article/**Supplementary Material**. Further inquiries can be directed to the corresponding authors.

## Supplementary material

Supplementary Figure 1 Analytical validation of the digital urine physicalomics pipeline demonstrates high precision and systemic agreement. **(a, b)**, Bar charts displaying the intra-assay repeatability **(a)** and inter-assay intermediate precision **(b)** of the digital physicalomics measurements. The coefficients of variation (CV, %) were evaluated for both Foam Half-life (*T*_1/2_, blue bars) and Chromaticity (*b**, orange bars) across three distinct clinical specific gravity (SG) stratifications (Low SG, Medium SG, and High SG) to ensure broad physiological applicability. Individual data points represent independent analytical runs. The horizontal red dashed line denotes the established clinical acceptability threshold for novel biomarkers (CV = 5.0%). Both physical parameters consistently maintained CVs strictly below this threshold across all concentration ranges. Error bars indicate the mean ± s.d. **(c, d)**, Bland-Altman plots evaluating the absolute measurement agreement across paired technical replicates for Foam Half-life **(c)** and Chromaticity **(d)**. The solid central lines indicate the mean systematic bias (+0.15 seconds for *T*_1/2_ and -0.08 units for *b**). The upper and lower dashed lines denote the 95% limits of agreement (+1.96 s.d. and -1.96 s.d., respectively). Both macroscopic features exhibit exceptional systemic agreement with near-zero mean bias, and the scatter distributions demonstrate no proportional bias across the dynamic measurement ranges. CV, coefficient of variation; SG, specific gravity; s.d., standard deviation.

Supplementary Figure 2 Standalone diagnostic performance of digital tensiometry (foam half-life) for detecting pathological albuminuria. a, b, Receiver Operating Characteristic (ROC) analyses evaluating the independent discriminative capacity of the digital Foam Half-life (*T*_1/2_) for identifying established clinical albuminuria thresholds across the entire study cohort (N=364). **(a)**, Diagnostic performance for detecting microalbuminuria (Urinary Albumin-to-Creatinine Ratio [UACR] ≥ 30 mg/g). The standalone optical biomarker achieved an Area Under the Curve (AUC) of 0.81 (95% CI: 0.76–0.86). The solid point indicates the optimal Youden index cutoff of *T*_1/2_ > 14.5 seconds, yielding a sensitivity of 78.4% and a specificity of 80.2%. **(b)**, Diagnostic performance for detecting macroalbuminuria (UACR ≥ 300 mg/g). The discriminative accuracy was further pronounced for severe proteinuria, achieving an excellent AUC of 0.88 (95% CI: 0.83–0.93). The solid point indicates the optimal cutoff of *T*_1/2_ > 41.2 seconds, providing a high sensitivity of 85.1% and a specificity of 87.6%. In both panels, the grey dashed diagonal lines represent the line of random classification. **(c)**, Distribution of Foam Half-life (*T*_1/2_) stratified by clinical UACR severity stages: Normoalbuminuria (< 30 mg/g), Microalbuminuria (30–299 mg/g), and Macroalbuminuria (≥ 300 mg/g). The y-axis is log_10_-transformed for enhanced visualization of the long-tail data distribution. The horizontal dashed lines explicitly represent the optimal diagnostic thresholds derived from the ROC analyses in a and b (14.5 s and 41.2 s, respectively). This mapping visually demonstrates the precise stage-wise demarcation achieved by the digital tensiometric signature, validating its clinical utility as a rapid, zero-reagent optical triage tool for evaluating pathological protein excretion.

Supplementary Figure 3 Systemic and urinary metabolic landscapes reveal distinct DKD-associated signatures and robust multivariate discrimination. **(a, b)**, Orthogonal Partial Least Squares Discriminant Analysis (OPLS-DA) score plots demonstrating clear metabolic separation across the study cohorts in both serum **(a)** and urine **(b)**. Each point represents an individual participant: Healthy Controls (HC, blue circles, n=80), T2DM without Kidney Disease (T2DM-NoDKD, orange circles, n=102), and Diabetic Kidney Disease (DKD, red circles, n=100). The shaded ellipses denote the 95% confidence intervals for each group. Both models exhibited high goodness-of-fit and predictability (Serum: *R^2^Y* = 0.94, *Q*^2^ = 0.86; Urine: *R^2^Y* = 0.91, *Q*^2^ = 0.83). Model validity was rigorously confirmed via 1,000-iteration permutation tests, which yielded significantly negative *Q*^2^ y-intercepts (Serum *Q*^2^ intercept = -0.28; Urine *Q*^2^ intercept = -0.21), definitively excluding the possibility of mathematical overfitting. **(c)**, Volcano plot visualizing the differential metabolite expression profile between the DKD and T2DM-NoDKD groups. The x-axis represents the log_2_ fold change (FC), and the y-axis represents the -log_10_ (FDR-adjusted P-value). The horizontal and vertical dashed lines denote the prespecified statistical thresholds for significance (FDR< 0.05 and |log_2_ FC| > 1.0, respectively). Metabolites significantly upregulated in DKD are highlighted in red, whereas those significantly downregulated are highlighted in blue. Non-significant features are shown in grey. Key pathophysiological markers, including protein-bound uremic toxins (Indoxyl sulfate, p-Cresyl sulfate), long-chain acylcarnitines (C16-, C18-Carnitine), and depleted TCA cycle intermediates (Succinate, Citrate), are explicitly annotated. **(d)**, Kyoto Encyclopedia of Genes and Genomes (KEGG) metabolic pathway enrichment analysis derived from the significant differential metabolites. The x-axis indicates the topological Pathway Impact score (ranging from 0 to 1). Bubble size is strictly proportional to the number of significantly altered metabolites hitting a specific pathway (Metabolite Count). The color gradient from blue to dark red indicates increasing statistical significance based on unadjusted -log_10_ P-values from hypergeometric testing. The analysis reveals profound perturbations in amino acid metabolism, fatty acid degradation, and cellular energetic pathways (TCA cycle, Glycolysis) driving the DKD metabotype. Statistical note: Differential expression in panel c was assessed using a two-sided Mann-Whitney U test, with P-values corrected for multiple comparisons utilizing the Benjamini-Hochberg false discovery rate (FDR) procedure. DKD, diabetic kidney disease; T2DM-NoDKD, type 2 diabetes mellitus without kidney disease; HC, healthy controls; PAG, phenylacetylglutamine; FDR, false discovery rate; FC, fold change.

Supplementary Table 1 Liquid Chromatography-Tandem Mass Spectrometry (LC-MS/MS) Multiple Reaction Monitoring (MRM) parameters and analytical validation metrics for targeted protein-bound uremic toxins. Analytical validation was performed on a high-performance liquid chromatography system coupled to a triple quadrupole mass spectrometer (HPLC-QqQ-MS) operated in Multiple Reaction Monitoring (MRM) mode. * Collision Energy (CE) values are presented for the quantifier and qualifier product ions, respectively. Analytical Limits: The Limit of Detection (LOD) is defined at a signal-to-noise (*S/N*) ratio of ≥ 3. The Limit of Quantification (LOQ) is defined at *S/N* ≥ 10 with a precision of<15% Coefficient of Variation (CV). All clinical samples analyzed in this study yielded concentrations robustly above the established LOQ. ESI (-), Electrospray Ionization in negative mode; *m/z*, mass-to-charge ratio; *t_R_*, retention time; CE, collision energy; SIL-IS, Stable Isotope-Labeled Internal Standard; LOD, Limit of Detection; LOQ, Limit of Quantification; MSI, Metabolomics Standards Initiative.

Supplementary Table 2 Sensitivity analysis of the FluxPro-DKD Fusion Score predicting composite adverse renal outcomes, accounting for the competing risk of non-renal mortality. Primary Endpoint: The composite adverse renal outcome, defined as progression to end-stage renal disease (ESRD), initiation of renal replacement therapy, or a sustained ≥ 40% decline in eGFR from baseline. Competing Event: Non-renal mortality prior to the renal endpoint, predominantly driven by cardiovascular death. In the standard Cox model, patients experiencing non-renal mortality were right-censored at the time of death. In the Fine-Gray model, non-renal mortality was explicitly treated as a competing risk to prevent informative censoring bias and the overestimation of absolute renal risk. *Statistical observation: Note the distinct attenuation of the age covariate in the Fine-Gray model compared to the standard Cox model. This is biologically expected, as advancing age is heavily associated with the competing risk of cardiovascular mortality, highlighting the rigorous necessity of the subdistribution approach in this advanced DKD cohort. csHR, cause-specific hazard ratio; sHR, subdistribution hazard ratio; CI, confidence interval; eGFR, estimated glomerular filtration rate (ml/min/1.73m^2^); UACR, urinary albumin-to-creatinine ratio (*mg/g*); HbA1c, glycated hemoglobin.
