# Supplementary material for: Integration of digital physicalomics and dual-fluid metabolomics flux ratios reveals tubular secretory dysfunction in early diabetic kidney disease
Source: Front Endocrinol (Lausanne). 2026 Apr 22;17:1802447. doi: 10.3389/fendo.2026.1802447 (PMC13143680; doi:10.3389/fendo.2026.1802447)
Supplement: Supplementary Figure 1 — Analytical validation of the digital urine physicalomics pipeline demonstrates high precision and systemic agreement. (a, b), Bar charts displaying the intra-assay repeatability (a) and inter-assay intermediate precision (b) of the digital physicalomics measurements. The coefficients of variation (CV, %) were evaluated for both Foam Half-life (T1/2, blue bars) and Chromaticity (b*, orange bars) across three distinct clinical specific gravity (SG) stratifications (Low SG, Medium SG, and High SG) to ensure broad physiological applicability. Individual data points represent independent analytical runs. The horizontal red dashed line denotes the established clinical acceptability threshold for novel biomarkers (CV = 5.0%). Both physical parameters consistently maintained CVs strictly below this threshold across all concentration ranges. Error bars indicate the mean ± s.d. (c, d), Bland-Altman plots evaluating the absolute measurement agreement across paired technical replicates for Foam Half-life (c) and Chromaticity (d). The solid central lines indicate the mean systematic bias (+0.15 seconds for T1/2 and -0.08 units for b*). The upper and lower dashed lines denote the 95% limits of agreement (+1.96 s.d. and -1.96 s.d., respectively). Both macroscopic features exhibit exceptional systemic agreement with near-zero mean bias, and the scatter distributions demonstrate no proportional bias across the dynamic measurement ranges. CV, coefficient of variation; SG, specific gravity; s.d., standard deviation. [file Supplementaryfile1.docx]

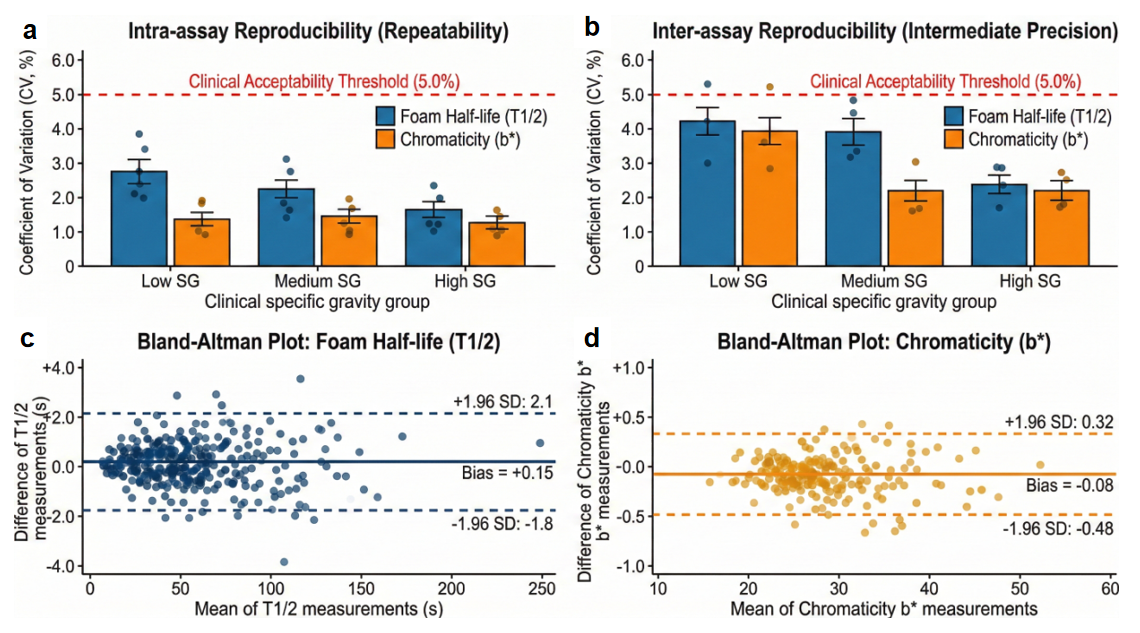


**Supplementary Fig. 1 | Analytical validation of the digital urine physicalomics pipeline demonstrates high precision and systemic agreement.**

a, b, Bar charts displaying the intra-assay repeatability (a) and inter-assay intermediate precision (b) of the digital physicalomics measurements. The coefficients of variation (CV, %) were evaluated for both Foam Half-life (*T*_1/2_, blue bars) and Chromaticity (*b**, orange bars) across three distinct clinical specific gravity (SG) stratifications (Low SG, Medium SG, and High SG) to ensure broad physiological applicability. Individual data points represent independent analytical runs. The horizontal red dashed line denotes the established clinical acceptability threshold for novel biomarkers (CV = 5.0%). Both physical parameters consistently maintained CVs strictly below this threshold across all concentration ranges. Error bars indicate the mean ± s.d.

c, d, Bland-Altman plots evaluating the absolute measurement agreement across paired technical replicates for Foam Half-life (c) and Chromaticity (d). The solid central lines indicate the mean systematic bias (+0.15 seconds for *T*_1/2_ and -0.08 units for *b**). The upper and lower dashed lines denote the 95% limits of agreement (+1.96 s.d. and -1.96 s.d., respectively). Both macroscopic features exhibit exceptional systemic agreement with near-zero mean bias, and the scatter distributions demonstrate no proportional bias across the dynamic measurement ranges. Abbreviations: CV, coefficient of variation; SG, specific gravity; s.d., standard deviation.


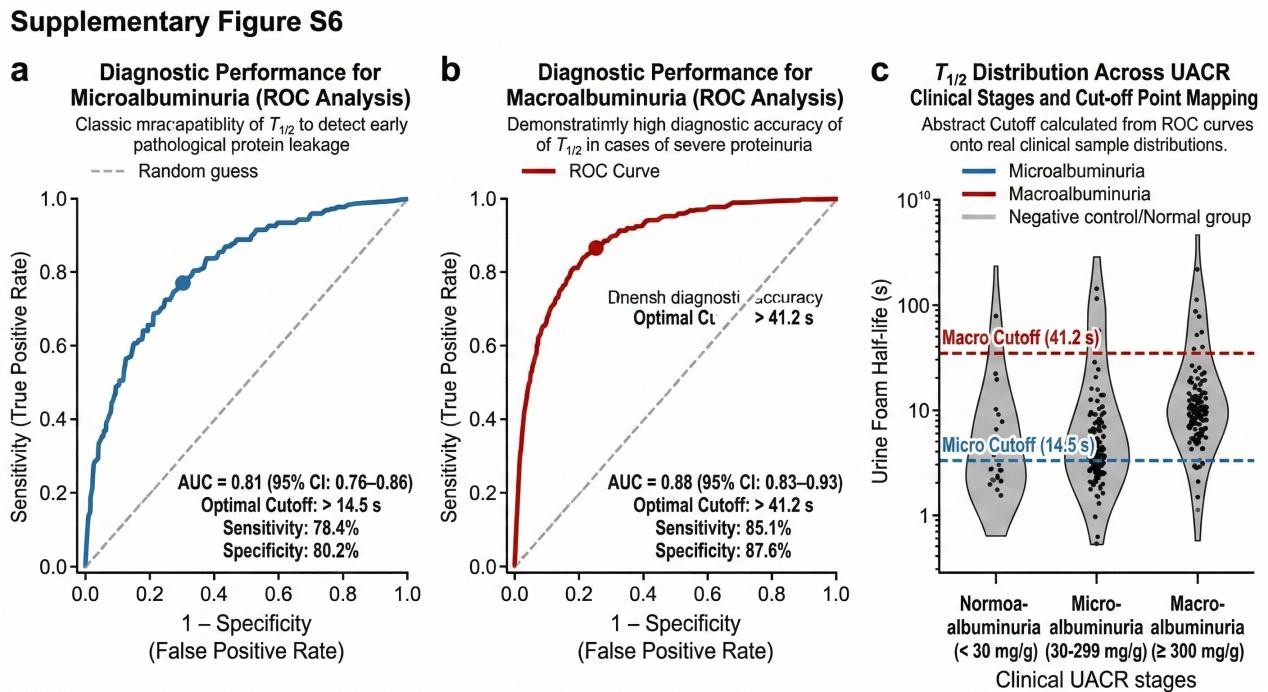


**Supplementary Fig. 2 | Standalone diagnostic performance of digital tensiometry (foam half-life) for detecting pathological albuminuria.**

a, b, Receiver Operating Characteristic (ROC) analyses evaluating the independent discriminative capacity of the digital Foam Half-life (*T*_1/2_) for identifying established clinical albuminuria thresholds across the entire study cohort (N=364). a, Diagnostic performance for detecting microalbuminuria (Urinary Albumin-to-Creatinine Ratio [UACR] ≥ 30 mg/g). The standalone optical biomarker achieved an Area Under the Curve (AUC) of 0.81 (95% CI: 0.76–0.86). The solid point indicates the optimal Youden index cutoff of *T*_1/2_ > 14.5 seconds, yielding a sensitivity of 78.4% and a specificity of 80.2%. b, Diagnostic performance for detecting macroalbuminuria (UACR ≥ 300 mg/g). The discriminative accuracy was further pronounced for severe proteinuria, achieving an excellent AUC of 0.88 (95% CI: 0.83–0.93). The solid point indicates the optimal cutoff of *T*_1/2_ > 41.2 seconds, providing a high sensitivity of 85.1% and a specificity of 87.6%. In both panels, the grey dashed diagonal lines represent the line of random classification.

c, Distribution of Foam Half-life (*T*_1/2_) stratified by clinical UACR severity stages: Normoalbuminuria (< 30 mg/g), Microalbuminuria (30–299 mg/g), and Macroalbuminuria (≥ 300 mg/g). The y-axis is log_10_-transformed for enhanced visualization of the long-tail data distribution. The horizontal dashed lines explicitly represent the optimal diagnostic thresholds derived from the ROC analyses in a and b (14.5 s and 41.2 s, respectively). This mapping visually demonstrates the precise stage-wise demarcation achieved by the digital tensiometric signature, validating its clinical utility as a rapid, zero-reagent optical triage tool for evaluating pathological protein excretion.


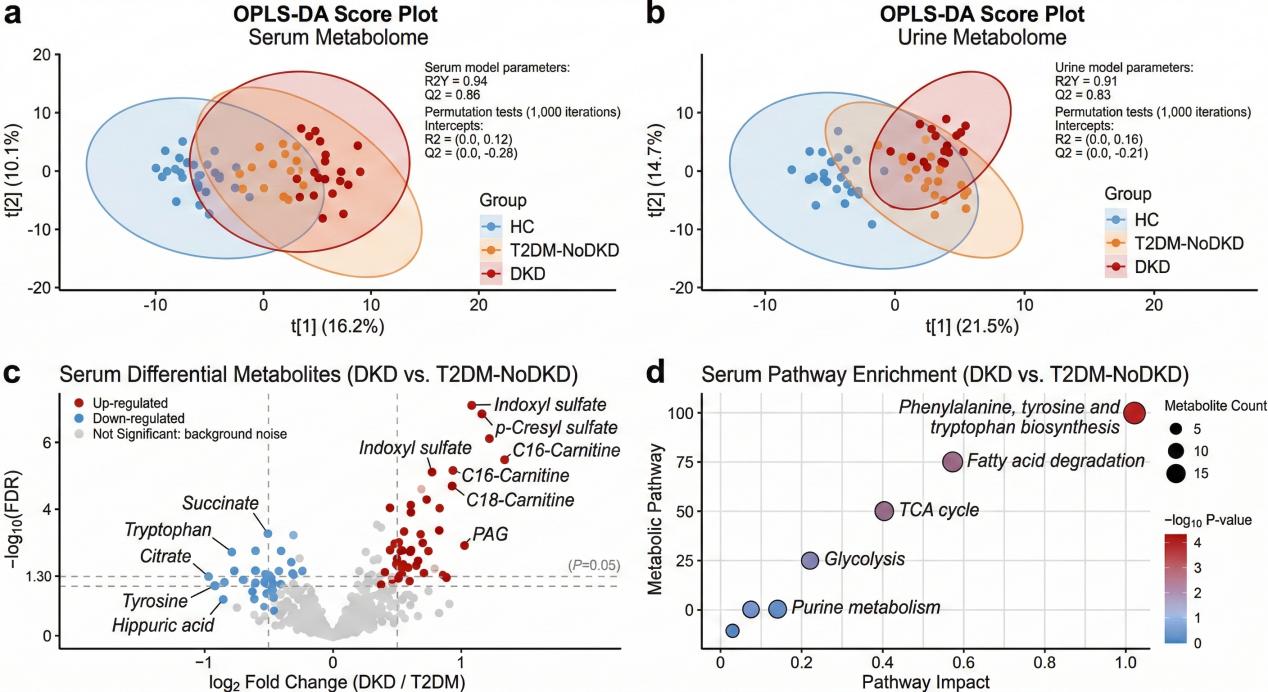


**Supplementary Fig. 3 | Systemic and urinary metabolic landscapes reveal distinct DKD-associated signatures and robust multivariate discrimination.**

a, b, Orthogonal Partial Least Squares Discriminant Analysis (OPLS-DA) score plots demonstrating clear metabolic separation across the study cohorts in both serum (a) and urine (b). Each point represents an individual participant: Healthy Controls (HC, blue circles, n=80), T2DM without Kidney Disease (T2DM-NoDKD, orange circles, n=102), and Diabetic Kidney Disease (DKD, red circles, n=100). The shaded ellipses denote the 95% confidence intervals for each group. Both models exhibited high goodness-of-fit and predictability (Serum: *R^2^Y* = 0.94, *Q*^2^ = 0.86; Urine: *R^2^Y* = 0.91, *Q*^2^ = 0.83). Model validity was rigorously confirmed via 1,000-iteration permutation tests, which yielded significantly negative *Q*^2^ y-intercepts (Serum *Q*^2^ intercept = -0.28; Urine *Q*^2^ intercept = -0.21), definitively excluding the possibility of mathematical overfitting.

c, Volcano plot visualizing the differential metabolite expression profile between the DKD and T2DM-NoDKD groups. The x-axis represents the log_2_ fold change (FC), and the y-axis represents the -log_10_ (FDR-adjusted P-value). The horizontal and vertical dashed lines denote the prespecified statistical thresholds for significance (FDR < 0.05 and |log_2_ FC| > 1.0, respectively). Metabolites significantly upregulated in DKD are highlighted in red, whereas those significantly downregulated are highlighted in blue. Non-significant features are shown in grey. Key pathophysiological markers, including protein-bound uremic toxins (Indoxyl sulfate, p-Cresyl sulfate), long-chain acylcarnitines (C16-, C18-Carnitine), and depleted TCA cycle intermediates (Succinate, Citrate), are explicitly annotated.

d, Kyoto Encyclopedia of Genes and Genomes (KEGG) metabolic pathway enrichment analysis derived from the significant differential metabolites. The x-axis indicates the topological Pathway Impact score (ranging from 0 to 1). Bubble size is strictly proportional to the number of significantly altered metabolites hitting a specific pathway (Metabolite Count). The color gradient from blue to dark red indicates increasing statistical significance based on unadjusted -log_10_ P-values from hypergeometric testing. The analysis reveals profound perturbations in amino acid metabolism, fatty acid degradation, and cellular energetic pathways (TCA cycle, Glycolysis) driving the DKD metabotype.

Statistical note: Differential expression in panel c was assessed using a two-sided Mann-Whitney U test, with P-values corrected for multiple comparisons utilizing the Benjamini-Hochberg false discovery rate (FDR) procedure. Abbreviations: DKD, diabetic kidney disease; T2DM-NoDKD, type 2 diabetes mellitus without kidney disease; HC, healthy controls; PAG, phenylacetylglutamine; FDR, false discovery rate; FC, fold change.

**Supplementary Table S1 | Liquid Chromatography-Tandem Mass Spectrometry (LC-MS/MS) Multiple Reaction Monitoring (MRM) parameters and analytical validation metrics for targeted protein-bound uremic toxins.**

| Metabolite | Ion Mode | Precursor Ion (m/z) | Quantifier Ion (m/z) | Qualifier Ion (m/z) | CE (eV)* | tR​ (min) | Internal Standard (SIL-IS) | LOD (nmol/L) | LOQ (nmol/L) | MSI Level |
| --- | --- | --- | --- | --- | --- | --- | --- | --- | --- | --- |
| Indoxyl Sulfate (IS) | ESI (-) | 212 | 80 | 132 | 25 / 15 | 3.45 | 13C6​-Indoxyl Sulfate | 15.2 | 45.5 | Level 1 |
| p-Cresyl Sulfate (pCS) | ESI (-) | 187 | 107 | 80 | 20 / 30 | 4.12 | d7​-p-Cresyl Sulfate | 22.4 | 67 | Level 1 |
| 13C6​-Indoxyl Sulfate | ESI (-) | 218 | 80 | — | 25 | 3.45 | — (Reference IS) | — | — | — |
| d7​-p-Cresyl Sulfate | ESI (-) | 194 | 114 | — | 20 | 4.12 | — (Reference IS) | — | — | — |

Notes: Analytical validation was performed on a high-performance liquid chromatography system coupled to a triple quadrupole mass spectrometer (HPLC-QqQ-MS) operated in Multiple Reaction Monitoring (MRM) mode.

* Collision Energy (CE) values are presented for the quantifier and qualifier product ions, respectively.

Analytical Limits: The Limit of Detection (LOD) is defined at a signal-to-noise (*S/N*) ratio of ≥ 3. The Limit of Quantification (LOQ) is defined at *S/N* ≥ 10 with a precision of <15% Coefficient of Variation (CV). All clinical samples analyzed in this study yielded concentrations robustly above the established LOQ. Abbreviations: ESI (-), Electrospray Ionization in negative mode; *m/z*, mass-to-charge ratio; *t_R_*, retention time; CE, collision energy; SIL-IS, Stable Isotope-Labeled Internal Standard; LOD, Limit of Detection; LOQ, Limit of Quantification; MSI, Metabolomics Standards Initiative.

**Supplementary Table S2 | Sensitivity analysis of the FluxPro-DKD Fusion Score predicting composite adverse renal outcomes, accounting for the competing risk of non-renal mortality.**

| Variables | Standard Cox Proportional Hazards Model |  |  | Fine-Gray Subdistribution Hazard Model |  |  |
| --- | --- | --- | --- | --- | --- | --- |
|  | csHR (95% CI) | Z-statistic | P value | sHR (95% CI) | Z-statistic | P value |
| FluxPro-DKD Fusion Score |  |  |  |  |  |  |
| Quartile 1 (Lowest) | 1.00 (Reference) | — | — | 1.00 (Reference) | — | — |
| Quartile 2 | 1.35 (0.85–2.10) | 1.28 | 0.184 | 1.30 (0.82–2.05) | 1.15 | 0.221 |
| Quartile 3 | 1.88 (1.25–2.85) | 3.01 | 0.003 | 1.80 (1.18–2.75) | 2.75 | 0.006 |
| Quartile 4 (Highest) | 2.85 (1.92–4.23) | 5.22 | <0.001 | 2.68 (1.81–3.96) | 4.85 | <0.001 |
| Clinical Covariates |  |  |  |  |  |  |
| Age (per 10-year increase) | 1.12 (0.95–1.32) | 1.35 | 0.152 | 1.04 (0.88–1.23)* | 0.46 | 0.645 |
| Sex (Male vs. Female) | 1.22 (0.92–1.62) | 1.38 | 0.165 | 1.18 (0.88–1.58) | 1.1 | 0.27 |
| Baseline eGFR (per 10 unit decrease) | 1.45 (1.22–1.72) | 4.25 | <0.001 | 1.41 (1.18–1.68) | 3.82 | <0.001 |
| log10​(UACR) (per 1-unit increase) | 1.65 (1.35–2.02) | 4.88 | <0.001 | 1.60 (1.30–1.97) | 4.45 | <0.001 |
| HbA1c (per 1% increase) | 1.15 (1.02–1.30) | 2.3 | 0.021 | 1.13 (1.01–1.28) | 2.15 | 0.035 |

Primary Endpoint: The composite adverse renal outcome, defined as progression to end-stage renal disease (ESRD), initiation of renal replacement therapy, or a sustained ≥ 40% decline in eGFR from baseline. Competing Event: Non-renal mortality prior to the renal endpoint, predominantly driven by cardiovascular death. In the standard Cox model, patients experiencing non-renal mortality were right-censored at the time of death. In the Fine-Gray model, non-renal mortality was explicitly treated as a competing risk to prevent informative censoring bias and the overestimation of absolute renal risk. *Statistical observation: Note the distinct attenuation of the age covariate in the Fine-Gray model compared to the standard Cox model. This is biologically expected, as advancing age is heavily associated with the competing risk of cardiovascular mortality, highlighting the rigorous necessity of the subdistribution approach in this advanced DKD cohort. Abbreviations: csHR, cause-specific hazard ratio; sHR, subdistribution hazard ratio; CI, confidence interval; eGFR, estimated glomerular filtration rate (ml/min/1.73m^2^); UACR, urinary albumin-to-creatinine ratio (*mg/g*); HbA1c, glycated hemoglobin.
